# Antioxidant Defence in *Labeo rohita* to Biotic and Abiotic Stress: Insight from mRNA Expression, Molecular Characterization and Recombinant Protein-Based ELISA of Catalase, Glutathione Peroxidase, CuZn Superoxide Dismutase, and Glutathione S-Transferase

**DOI:** 10.3390/antiox13010018

**Published:** 2023-12-21

**Authors:** Sonali Parida, Pramoda Kumar Sahoo

**Affiliations:** ICAR-Central Institute of Freshwater Aquaculture, Kausalyaganga, Bhubaneswar 751002, India; paridasonali08@gmail.com

**Keywords:** catalase (CAT), CuZn superoxide dismutase (CuZnSOD), glutathione peroxidase-1 (GPX-1), glutathione S-transferase-mu (GST-mu), *Labeo rohita*, antimicrobial activity

## Abstract

Fish possess numerous enzymatic antioxidant systems as part of their innate immunity. These systems have been poorly studied in *Labeo rohita* (rohu). The present study characterized and investigated the role of antioxidant genes in the defence mechanisms against two types of stressors, including infection and ammonia stress. Four key genes associated with antioxidant activity–catalase, glutathione peroxidase, glutathione S-transferase, and CuZn superoxide dismutase were successfully cloned and sequenced. These genes were found to be expressed in different tissues and developmental stages of rohu. The expression levels of these antioxidant genes in the liver and anterior kidney tissues of rohu juveniles were modulated in response to bacterial infection (*Aeromonas hydrophila*), parasite infection (*Argulus siamensis*), poly I:C stimulation and ammonia stress. Additionally, the recombinant proteins derived from these genes exhibited significant antioxidant and antibacterial activities. These proteins also demonstrated a protective effect against *A. hydrophila* infection in rohu and had an immunomodulatory role. Furthermore, indirect ELISA assay systems were developed to measure these protein levels in healthy as well as *A. hydrophila* and ammonia-induced rohu serum. Overall, this study characterized and emphasised the importance of the antioxidant mechanism in rohu’s defence against oxidative damage and microbial diseases.

## 1. Introduction 

The unfavourable conditions caused by hazardous chemicals and continued exposure to endemic pathogens lead to several metabolic changes in host tissue, foremost of which is the production of reactive oxygen species (ROS) or free radicals through the univalent reduction of O_2_. These ROS include hydroxyl radicals, superoxide, and hydrogen peroxide [1]. The presence of highly reactive chemicals in the host organism can lead to several detrimental effects, including DNA hydroxylation, protein denaturation, apoptosis, lipid peroxidation, and, ultimately, cellular death [2]. Nevertheless, the cell’s robust antioxidant defences neutralize these free radicals without noticeable adverse consequences. The antioxidant defence system is predominantly comprised of the enzymatic actions of superoxide dismutase, glutathione peroxidase, catalase, glutathione S-transferase, and peroxiredoxin [3,4].

Extensive research has been conducted on enzymatic antioxidants with the aim of mitigating and managing diseases that arise as a consequence of oxidative damage. Catalase, an antioxidant enzyme, is widely present in oxygen-respiring organisms and plays a critical role in maintaining the equilibrium between the production and elimination of hydrogen peroxide (H_2_O_2_) and is also vital for the proper functioning of innate immunity [5,6,7]. The previous study explored catalase’s involvement in supporting a key immune mechanism in the Drosophila digestive tract, underscoring its importance in the non-specific defence system of invertebrates [6,8]. Additionally, significant expression of the catalase gene in haemocytes of *Chilo suppressalis* a Lepidoptera suggests its role in innate defence [9]. In *Caenorhabditis elegans*, stimulation of catalase leads to the up-regulation of the antimicrobial gene, indicating its contribution to innate immunity and immunomodulation [10]. The presence of catalase is documented in various fish species, exhibiting its conserved sequence and functional domains [11,12,13,14,15,16,17]. The up-regulation of catalase expression has been seen in response to several stimuli, including bacterial and viral infections, temperature stress, and exposure to toxic substances [18,19,20]. 

Glutathione peroxidase (GPx) is a crucial intracellular enzyme involved in antioxidant defense, and operates primarily within mitochondria and also within the cytosol [21]. Its primary function is to catalyze the breakdown of hydrogen peroxide (H_2_O_2_) into water and lipid peroxides into their respective alcohols. The enzymatic function of GPx is reliant on a micronutrient cofactor called selenium, leading to its name as selenocysteine peroxidase [22]. GPx is vital for protecting cells against oxidative stress inhibiting lipid peroxidation [23]. Humans possess eight distinct GPx enzymes (GPx1– GPx8), which are located on chromosomes 3, 14, 5, 19, 6, 6, 1, and 5, respectively [24]. According to Drevet (2006) [25], GPx-1 is the predominant selenoperoxidase found in nearly all cellular types. Numerous studies underscore the significance of GPx1 in the context of many diseases. In a prior study, Forgione et al. (2002) [26] postulated that the absence of GPx1 leads to a direct elevation in vascular oxidative stress, resulting in damage to endothelial cells. Furthermore, the GPx gene has been extensively studied in diverse aquatic organisms, spanning various species [27,28,29,30,31,32,33]. According to Do et al. (2019) [27], the expression level of GPx1 in *Tor lambroid* indicates its involvement in the oxidative burst triggered by temperature stress. The GPx-gene-based DNA vaccine was administered to orange-spotted grouper *(Epinephelus coioides*) that were subsequently exposed to *Vibrio harveyi.* The results indicate a significant increase in the relative percentage of survival (RPS), reaching as high as 77.5% [34].

Superoxide dismutase (SOD) is an additional potent antioxidant enzyme that functions by facilitating the dismutation of two superoxide anion (O_2_) molecules into hydrogen peroxide (H_2_O_2_) and molecular oxygen (O_2_), thereby reducing the potential harm caused by the superoxide anion [35,36]. SOD is categorized into three forms based on the presence of metal cofactors, such as iron (Fe), zinc (Zn), copper (Cu), and manganese (Mn). The forms of superoxide dismutase (SOD) that are commonly observed in prokaryotes and certain plants’ chloroplasts are Fe-SOD. Mn-SOD, on the other hand, is found in prokaryotes and the mitochondria of eukaryotes. Cu/Zn-SOD, predominantly distributed in eukaryotes, is primarily localised in the cytosol, with an additional presence in chloroplasts and peroxisomes [37]. The gene CuZnSOD, which has been previously identified and located on chromosome 21 [38], has been extensively characterized in various aquatic organisms [15,39,40,41,42,43,44,45]. The significance of CuZnSOD expression in the non-specific bacterial defence, such as *Vibrio parahaemolyticus* in *Macrobrachium rosenbergii* [40], *Micrococcus luteus* in *Chlamys farreri* [42], and *Vibrio alginolyticus* in *Pseudosciana crocea* [46], has been demonstrated. The involvement of CuZnSOD has also been examined in the context of abiotic stressors, such as thermal stress in *Onychostoma macrolepis* [15], exposure to various chemicals, including polybrominated diphenyl ethers (PDBEs), in *Anodonta woodiana* [41], and the exposure of *Haliotis discus discus* to heavy metals such as cadmium, zinc and copper [47]. 

The glutathione S-transferase (GST) superfamily includes a class of compact proteins of 200–250 amino acids that are mobilized inside the organism to defend against oxidative stress and exposure to toxins [48]. The involvement of GSTs is of utmost significance in the process of eliminating hydrophobic xenobiotics. This is achieved through the catalytic process of conjugating reduced glutathione (GSH) with xenobiotics, thereby enhancing their solubility [49,50]. GSTs exhibit immunomodulatory properties and have potential for vaccine development [51]. They also play a significant role in the repair of damaged macromolecules. Moreover, they serve as biomarkers for detecting alterations in the environment at the biochemical level [52]. GSTs are categorized into three discrete types based on their cellular localization, namely cytosolic, mitochondrial, and microsomal [48]. The cytosolic class is involved in cellular detoxification and has seven subclasses, namely Delta, Epsilon, Omega, Sigma, Theta, Mu, and Zeta. The immunological role of GST has been investigated in various aquatic organisms [53,54,55,56,57]. The GST Mu in *M. rosenbergii* exhibits functional activity against bacterial infections caused by *Vibrio anguillarum* in *M. rosenbergii* [58].

The metabolic interplays of malicious factors resulting in oxidative stress and the antioxidant mechanisms in place to combat them quite predictably attain the utmost importance for mankind when they affect global commercial aquaculture dynamics. *L. rohita* is a highly preferred carp and fetches comparatively high market prices. Its global production has touched 2.48 MMT, contributing to 5.1% of total inland aquaculture production [59]. Diseases caused by the bacteria *Aeromonas hydrophila* and the parasite *Argulus siamensis* are the major threats to this carp aquaculture [60,61]. Moreover, the important role of antioxidant enzymes during these infections has already been discussed in earlier studies [62,63]. The present study aims to investigate and understand the role of the four key antioxidant enzymes, i.e., catalase, GPx-1, CuZnSOD and GST-mu, in *L. rohita* during different biotic stress conditions induced by *A. hydrophila*, *A. siamensis*, dsRNA synthetic analogue poly I:C stimulation, as well as abiotic stress induced by ammonia toxicity. For this investigation, we have cloned, sequenced, and characterized these antioxidant genes and their recombinant proteins. ELISA-based assays were developed to detect their levels in *L. rohita*. Additionally, it is hypothesized that the levels of these antioxidants can be reliably detected and quantified using ELISA-based assays, providing a valuable tool for assessing the antioxidant response in fish under various stress conditions.

## 2. Material and Methods

### 2.1. Maintenance of L. rohita

The rohu juveniles (weight range: 25–30 g at six months of age) were obtained from the ICAR-Central Institute of Freshwater Aquaculture, located in Kausalyaganga, Bhubaneswar. A total of 420 fish were subjected to acclimatization in 500-litre tanks made of fibre-reinforced plastic (30 fish in each tank), equipped with an appropriate aeration system, within a wet laboratory setting for a duration of three weeks. The fish were provided with commercial pellet feed at 3% of their body weight twice daily. Regular monitoring was conducted to assess the water quality parameters, including temperature (ranging from 28.0 to 30.5 °C), dissolved oxygen levels (5.50 ± 0.43 mg/L), pH levels (7.7 ± 0.41), nitrite concentrations (0.02 ± 0.01 mg/L), and ammonia concentrations (0.10 ± 0.02 mg/L). The fish were subjected to anaesthesia using MS222 (tricaine methane sulfonate) (Sigma-Aldrich, St. Louis, MO, USA, Catalogue No. E10521-50G) for the purpose of immobilization during every procedure. Subsequently, the fish were euthanized by administering an excessive dose of the aforementioned anaesthetic agent for the purpose of obtaining samples. The tests were conducted in accordance with the standards of the Committee for the Purpose of Control and Supervision of Tests on Animals (CPCSEA) of the Government of India, with the consent of the Institute Animal Ethics Committee.

For the ontogeny study, adult *L. rohita* (weighing 1.50–2.00 kg) from one full-sib family used in the ongoing selective breeding programme were collected from the farm of the Central Institute of Freshwater Aquaculture, Bhubaneswar, India. For the ontogenic study, three pairs of sexually mature female and male rohu were taken. The three pairs of fish were bred separately following the standard induced breeding technique using “Ovaprim” (Biomeda MTC Animal Health, Cambridge, ON, Canada). Eggs were collected from each female by stripping after 6 h of the hormone injection. The males were stripped individually to collect sperm, and the same was used to fertilize eggs collected from individual females for external fertilization. The fertilized eggs from individual pairs were separately hatched in a glass-jar hatchery and maintained in FRP tanks of 40 L for up to 15 days using the standard procedure under plankton, and artificial powdered spawn feed provided ad libitum with daily change of water of tanks. Approximately 50–100 mg of sperm, eggs and young ones were collected for RNA isolation separately from individual sets of broods. For tissue-specific expression analysis of antioxidant genes, three juveniles were sacrificed, as detailed later, and the tissue samples were collected aseptically for RNA isolation, as described later. 

To study the effect of biotic stressors (*Aeromonas hydrophila* infection), twenty fish were maintained in three FRP tanks and subjected to a bacterial challenge, as detailed later. Three fish (one from each tank at each time point) were sampled for the collection of liver and anterior kidney tissues at ten different time points post-challenge, as detailed later in Section 2.4.2. To look into the expression pattern of the antioxidant genes following parasitic (*Argulus siamensis)* infection, six fish were maintained in four tanks and exposed to parasitic infection, as detailed later in Section 2.4.3. The liver and anterior kidney tissues were collected pre-challenge and post-challenge (at five points) from three different fish at each time point. Similarly, for poly I:C induction, ten fish each were maintained in three tanks separately (as detailed later in Section 2.4.4) and three fish were sampled (one from each tank) per time point for RNA isolation from liver and anterior kidney tissues. Further, twenty fish each in three tanks were used for ammonia (abiotic) stress and subsequent sample collection at ten different points (one fish from each tank at each time point), as detailed in Section 2.4.5.

For the immunomodulation study using recombinant antioxidant proteins, thirteen FRP tanks were used. Each tank contained ten fish (except five fish in the naïve control group). For each recombinant protein or PBS-control injection (as detailed in Section 2.9), three tanks were used.

### 2.2. RNA Isolation, cDNA Synthesis and PCR Amplification

Samples of liver and anterior kidney tissues from *Labeo rohita* were collected and preserved in RNAlater solution. The extraction of total RNA (50–100 mg) was carried out using TRI reagent (Sigma, St. Louis, MO, USA) following the instructions provided by the manufacturer. The RNA that had been isolated was subsequently subjected to treatment with DNase I, RNase-free (Fermentas, ThermoFisher Scientific, Wilmington, DE, USA). This treatment was performed in order to deactivate any remaining DNA residue present in the pure RNA samples, following the guidelines provided by the manufacturer. The RNA concentration was performed by measuring the absorbance at a wavelength of 260 nm. The assessment of sample purity was conducted by quantifying the OD260 nm to OD280 nm ratio using the NanoDrop ND1000 instrument (Thermo Scientific, Wilmington, DE, USA), with an anticipated range of values falling between 1.9 and 2.0. The assessment of purity was subsequently conducted using β-actin PCR, as described in a later study by Kar et al. (2016) [64]. To perform cDNA synthesis from total RNA, the Thermo Scientific Verso cDNA synthesis kit (ThermoFischer Scientific Inc., Graiciuno 8, LT-. 02241 Vilnius, Lithuania, USA) was employed in accordance with the manufacturer’s guidelines. This kit facilitated the generation of complementary DNA from 1 µg of total RNA through the process of reverse transcription. The complementary DNA (cDNA) product was stored at a temperature of −20 °C in order to be utilized in further amplification procedures.

### 2.3. Cloning and Characterization of Catalase (LrCAT), CuZnSOD (LrCuZnSOD), GPx1 (Lr GPx1) and GST-mu (LrGST-mu) mRNA of L. rohita 

For each of the antioxidant genes in *L. rohita*, i.e., catalase (*Lr*CAT), CuZnSOD (*Lr*CuZnSOD), GPx1 (*Lr*GPx1), and GST-mu (*Lr*GST-mu), a primer set consisting of a pair of full CDs was designed. These primer sets were developed using the consensus antioxidant sequences obtained from a limited number of sequences available for teleosts in the NCBI database. The primer design software used in this study was Primer Premier 5 (version 5.0, Premier Biosoft International, Palo Alto, CA, USA). The synthesis of the primers was conducted by Integrated DNA Technologies (Coralville, IA, USA), as indicated in Table 1. The genes encoding antioxidants were subjected to amplification using polymerase chain reaction (PCR) under specific conditions. The PCR protocol comprised an initial denaturation step at 95 °C for 2 min, followed by denaturation at 95 °C for 45 s. The annealing step was performed at different temperatures depending on the gene being amplified: 52 °C for catalase, 56 °C for CuZnSOD, GPx1, and GST-mu, each for 45 s. The extension step was carried out at 72 °C for 1 min and 30 s, and this entire cycle was repeated 35 times. Finally, a final extension step was performed at 72 °C for 10 min. The details of the PCR conditions are summarized in Table 1. The PCR products were analysed in agarose (1%) gel and subsequent purification of the amplicons was conducted with a gel purification kit provided by Bangalore Genei Pvt. Ltd. (Mumbai, India). The PCR amplicons that had been purified were inserted into a T-vector and introduced into competent *Escherichia coli* DH5α cells through transformation. This was achieved using the InsTAclone PCR Cloning Kit (Thermo Scientific, USA), following the instructions provided by the manufacturer. The putative clones were subsequently validated through DNA sequencing conducted by AgriGenome, India. The alignment of sequences was performed using the ClustalW multiple alignment tools of BioEdit version 7.0.0 [65]. The amino acid sequence was obtained by utilizing ORF Finder (http://www.ncbi.nlm.nih.gov/gorf/gorf.html, accessed on 25 July 2019) to derive the entire coding sequence. The signal peptide sequence was determined through the utilization of the Signal P bioinformatics tool (http://www.cbs.dtu.dk/services/SignalP/, accessed on 25 July 2019) [66]. The Compute PI/Mw program available on the ExPASy Bioinformatics Resource Portal (http://web.expasy.org/compute_pi/, accessed on 26 July 2019) was used to derive the isoelectric point and molecular weight of the mature proteins. The identification of functional domains was conducted through the utilization of SMART domain architecture analysis (http://smart.emblheidelberg.de/, accessed on 31 July 2019) and ExPASy Prosite (https://prosite.expasy.org/, accessed on 31 July 2019 ) as outlined by Gasteiger et al. (2003) [67] and Mohapatra et al. (2019) [61]. Furthermore, the analysis of the tertiary structure was conducted utilizing the ITASSER software [68]. The amino acid sequences of antioxidant proteins from different species were obtained from the NCBI database and compared to the amino acid sequences of *Lr*CAT, *Lr*CuZnSOD, *Lr*GPX1, and *Lr*GST-mu of *L. rohita* (Figures S1–S4). Phylogenetic analysis was conducted utilizing MEGA X.0 software version 10.2.6. The neighbour-joining approach was employed to generate phylogenetic trees, taking into account the evolutionary distance of antioxidant genes across several taxonomic groupings, including fish, amphibians, birds, reptiles, and mammals. Evolutionary distances are defined as the substitutions of the number of amino acids per site, as described by Tamura et al. (2013) [69]. The antimicrobial peptide (AMP) derived from the antioxidant sequence was analyzed using the AMPA web server (http://tcoffee.crg.cat/apps/ampa/do, accessed on 27 July 2019).

### 2.4. Expression Analysis of Antioxidant Genes

#### 2.4.1. Ontogeny and Tissue-Specific Expression 

In order to examine the transcription patterns of CAT, CuZnSOD, GPx-1, and GST-mu genes during ontogeny of *L. rohita*, specifically in milt, eggs, and larvae, samples were collected at various time points. These time points included 0, 1, 3, 6, 9, 12, 18, 24, 48, and 72 h post-fertilization (hpf), as well as 5 and 15 days post-fertilization (dpf). The collection of samples was carried out separately in three sets of broods, as described by Nayak et al. [73] in 2011. To examine the transcript levels of antioxidant genes in various tissues of healthy *L. rohita*, multiple tissue samples, including liver, muscle, skin, heart, kidneys (anterior and posterior), spleen, hindgut, foregut, brain, gills, and eye, were obtained from *L. rohita* juveniles. These samples were preserved in RNAlater and afterwards kept at a temperature of −20 °C for future analysis [66].

#### 2.4.2. Expression Analysis following Bacterial (*Aeromonas hydrophila*) Infection

The bacterial infection investigation utilised *A. hydrophila*, a Gram-negative bacteria belonging to the family Enterobacteriaceae [74]. The LD50 (lethal dosage 50) of the bacterium was determined using the methodology proposed by Reed and Muench in their study conducted in 1938 [75]. Moreover, a suspension of active *A. hydrophila* in 0.1 mL of phosphate-buffered saline (PBS) with a LD50 of 3 × 10^6^ colony forming units (cfu) per 0.1 mL of PBS per 20 gm of fish was administered through injection to a group of 60 *L. rohita* juvenile fish. A series of liver and kidney tissue samples were collected from fish that were subjected to a challenge. These samples were obtained at different time intervals, specifically at 0, 1, 3, 6, 12, 24, 48, and 72 h post-challenge (hpc), as well as 7 and 15 days post-challenge (dpc). The sampling was conducted in triplicate. The tissue samples were further processed for RNA isolation processes following Section 2.4.1.

#### 2.4.3. Expression Analysis following Parasitic (*Argulus siamensis*) Infection

As a parasite model, *A. siamensis*, a significant freshwater ectoparasite that mostly affects *L. rohita* (Strain-CIFA/AS/01), was used. Each *L. rohita* juvenile was challenged with 250 nos. of *A. siamensis* metanauplii [66]. There were four fish per group and six time points. They were then cautiously returned to the appropriate fibre-reinforced plastic (FRP) tanks. At intervals of five days, the parasite burden on each fish was examined visually [64]. Three fish were used for each time point, and liver and anterior kidney tissues were taken at different time points after the challenge (0, 12, 24 h, 3, 7, and 15 days) in RNAlater for further processing.

#### 2.4.4. Expression Analysis following Poly I:C Induction

To induce viral infection in juvenile *L. rohita*, the experimental approach involved the injection of Poly I:C (poly ionosinic: cytidylic), a synthetic double-stranded RNA (dsRNA) that mimics viral infection. A sample consisting of thirty rohu juveniles was selected for the purpose of this study. Each fish in the sample was administered an injection containing a dose of 500 µg/fish in 100 µL of PBS. The liver and anterior kidney tissues of the fish were sampled after stimulation with Poly I:C. These tissues were preserved in RNAlater at different time intervals (0, 1, 3, 6, 12, 24, 48, 72 h, and 7 and 15 days post-induction). Three fish were sampled at each time point, and the collected tissues were stored at −20 °C until further analysis.

#### 2.4.5. Expression Analysis following Ammonia-Induced Stress

Ammonia is one of the most well-known abiotic stressors for aquatic life. Using a static bioassay technique, the LC_50_ dose of ammonia was previously standardised and discovered to be 5 mg/L in *L. rohita* juveniles. Twenty numbers of naive fish raised in each of the three aquariums were used for ammonia stress using the LC_50_ dose of ammonia. At various time points (0, 1, 3, 6, 12, 24, 48, 72 h, and 7 and 15 d post-induction), the liver and anterior kidney tissues from control and infected fish were taken in triplicate in RNAlater for each time point as described earlier.

### 2.5. Relative Quantification by RT-PCR (qRT-PCR)

RNA was extracted and purified from the aforementioned RNAlater-preserved samples and subsequently subjected to cDNA synthesis using the previously described method. One specific primer set for each of the above-described antioxidant genes (Table 1) was designed using Primer Premier 5.0. The expressions of the antioxidant genes during different stress conditions were carried out using Light Cycler 96 SW 1.1, Roche, Germany [66]. In brief, a final reaction mixture of 10 μL was prepared, consisting of 5 μL of 2× Fast Start Essential DNA Green Master (manufactured by Roche, Roche Diagnostics GmbH Sandhofer Strasse 116 68305 Mannheim, Germany), 0.5 μL (equivalent to 5 pmole) of both forward and reverse primers, 20 ng of cDNA in 2 μL, and 2 μL of PCR grade H_2_O. The qRT-PCR protocol consisted of an initial pre-denaturation step at 95 °C for 10 min. The subsequent 40 cycles of amplification included denaturation at 95 °C for 10 s, annealing at 56 °C (for *Lr*CAT, *Lr*CuZnSOD, and *Lr*GPX-1) or 52 °C (for *Lr*GST-mu) for 10 s, and extension at 72 °C for 20 s. Later, a melt curve analysis was performed, involving temperature steps at 95 °C for 10 s, 65 °C for 60 s, and 97 °C for 1 s. Finally, the samples were cooled at 37 °C for 30 s. The qRT-PCR studies were conducted in triplicate for all samples, using β-actin as the reference gene, as described by Robinson et al. (2012) [70]. In the ontogeny study, the developmental stage of 0 h was examined, whereas in the study on tissue-specific expression, muscle tissue was selected as the calibrator for analysis. In all experiments pertaining to infection and induction, control samples taken at 0 h were utilised as study calibrators for the purpose of relative quantification, specifically for the calculation of 2^−ST-m^ [76]. 2^−275]^, where ΔΔCq = (ΔCq sample − ΔCq calibrator), was used to calculate the variance in expression pattern (fold difference) (Cq value of target gene–Cq value of reference genes). The analysis’s calibrator was the average Cq value of naive fish. The mean fold expression values were determined for each sample in triplicate.

### 2.6. Expression of Recombinant Proteins of LrCAT, LrCuZnSOD, LrGST-mu and LrGPX-1, Their Purification and Antibody Production

The genes responsible for encoding the proteins catalase (CAT), glutathione peroxidase (GPX-1), glutathione S-transferase (GST-mu), and CuZn superoxide dismutase (CuZnSOD) from the rohu species were artificially synthesised. This synthesis process took into account the codon preference of the bacterium *Escherichia coli* and utilised primers that contained restriction enzyme sites NdeI and XhoI at the 5’ and 3’ ends, respectively. The synthesized fragments were inserted into the pUC57 cloning vector at the EcoRV restriction site. The potential recombinants were subjected to analysis using restriction enzyme digestion, followed by confirmation through DNA sequencing. In order to conduct expression studies, the gene insert was subjected to purification from pUC57 using enzymatic digestion using NdeI and XhoI enzymes. Subsequently, the purified gene insert was cloned into the expression vector pET28a+, which had been digested using the same NdeI and XhoI enzymes. The recombinant samples were subjected to analysis by the process of restriction enzyme double digestion, after which the clones exhibiting positive results were chosen for further investigation.

The vector containing inserts was introduced into *E to induce expression. coli* BL21 (λDE3) cells. Protein expression was stimulated by the introduction of 1 mM IPTG at a final concentration once the culture reached an optical density of 0.6 at 600 nm. The cells that were stimulated were subsequently incubated for an additional 6 h at a temperature of 37 degrees Celsius. The SDS-PAGE examination revealed the presence of the anticipated protein bands of GPX-1 (16.6 kDa), CuZnSOD (18 kDa), GST-mu (24.31 kDa), and catalase (58.9 kDa). These findings were subsequently corroborated in Western blots; however, the corresponding data are not given here.

The production of antibodies against the relevant proteins was achieved by administering 200 μg of recombinant protein to rabbits using Freund’s complete adjuvant in a 1:1 ratio. Freund’s incomplete adjuvant was used to make booster injections of three doses, and then blood samples were obtained on day 30. Subsequently, the purification of the antibody was carried out following a standardised process. An enzyme-linked immunosorbent test (ELISA) using the above antibody was undertaken, as described later [66,77].

### 2.7. Antioxidant Activity of Recombinant Proteins as Measured through DNA Protection Assay 

The protective efficacy of the proteins r*Lr*CAT, r*Lr*CuZnSOD, r*Lr*GST-mu, and r*Lr*GPX-1 were then evaluated. The experiment aimed to investigate the ability of these proteins to safeguard the supercoiled plasmid DNA PBR322 against strand breakage, following a methodology previously described by Singh et al. (2009) [78] and Xu et al. (2012) [79], with slight modifications. In this experiment, plasmid DNA was subjected to UV irradiation for a duration of 10 min on the surface of a UV transilluminator (DUOVIEW, Genaxy Scientific, Himanchal Pradesh, India) with an intensity of 72 W, specifically at a wavelength of 254 nm while maintaining room temperature conditions along with a final concentration of hydrogen peroxide (H_2_O_2_) of 147 mM and varying quantities (6.25, 12.5, and 25 µg/mL) of r*Lr*CAT, r*Lr*CuZnSOD, r*Lr*GST-mu, and r*Lr*GPX-1. Additionally, positive control was included using BHA (butylated hydroxyl anisole), a chemical antioxidant. The level of oxidation was assessed after aduration of 30 min by subjecting the samples to electrophoresis on a 1% agarose gel (MP Biomedicals, 9 Goddard Irvine, CA 92618, USA). The ethidium bromide-stained gel was visualised in GelDoc (Bio-Rad Laboratories, 1000 Alfred Nobel Drive Hercules, California 94547 Inc. USA) [66].

### 2.8. Antimicrobial Activity of rLrCAT, rLrCuZnSOD, rLrGST-mu and rLrGPX-1

The study assessed the antibacterial effectiveness of r*Lr*GPX-1, r*Lr*GST-mu, r*Lr*CuZnSOD, and r*Lr*CAT proteins against two pathogenic bacterial strains, namely Gram-positive *Staphylococcus aureus* and Gram-negative *A. hydrophila* strains. These strains were obtained from our laboratory, and the minimal bactericidal concentration (MBC) assay was employed for evaluation. The MBC assay employed overnight cultures of *S. aureus* and *A. hydrophila*, with bacterial counts evaluated through measurement of optical density (OD) at a wavelength of 540 nm. The bacterial count at the end of the experiment was standardised to a concentration of 1 × 10^6^ colony-forming units per millilitre (cfu/mL) [66]. Various quantities of the recombinant protein in phosphate-buffered saline (PBS), namely 200, 100, 50, and 25 μg/mL, were generated alongside suitable control samples. The bacterial suspension was created and had a concentration of 1 × 10^6^ cfu/mL. This suspension was then combined with solutions of r*Lr*GPX-1, r*Lr*GST-mu, r*Lr*CuZnSOD, and r*Lr*CAT in the wells of sterile microplates. The microplates were thereafter incubated at a temperature of 37 °C. In addition, a 100 μL aliquot was evenly distributed onto tryptone soy agar plates at various time points (0, 3, 6, 12, and 24 h after incubation) and subsequently incubated at 37 °C overnight to quantify the colony count. The determination of antimicrobial activity, namely the minimum bactericidal concentration (MBC), was conducted by assessing the protein concentration at which 99% growth inhibition occurred, as described by Mohapatra et al. (2016) [72].

### 2.9. Immunomodulation Study Using Recombinant Antioxidant Proteins

The study utilised the relative percent survival (RPS) and assessed the immunomodulatory function of r*Lr*GPX-1, r*Lr*GST-mu, and r*Lr*CAT to evaluate the degree of protection against an experimental *A. hydrophila* challenge in rohu fish. Two groups of rohu juvenile fish, consisting of 30 individuals in each group, were subjected to intraperitoneal injections of either 0.1 mL of phosphate-buffered saline (PBS) as a control or *A. hydrophila* at a concentration of 5 × 10^6^ colony-forming units (cfu) per 0.1 mL of PBS per 20 g of body weight of the fish. These injections were administered following a 12 h period of intraperitoneal injection of r*Lr*GPX-1, r*Lr*GST-mu, and r*Lr*CAT proteins for each respective protein. In the study conducted by Mohapatra et al. (2016) [72], a group of five healthy fish were selected to serve as the control group. The calculation of RPS was performed in the following manner: The formula for calculating the relative percent survival (RPS) is derived from Amend’s work in 1991. It is expressed as follows: RPS = [1 − (% mortality of the experimental group/% mortality of the PBS-control group)] × 100. Additional kidney and liver tissues were obtained and preserved in RNAlater solution at various time intervals (0, 6, 12, 24, and 48 h) following the challenge. The samples were subsequently maintained at a temperature of −20 °C until they were ready for subsequent analysis. The samples underwent processing for RNA isolation and cDNA synthesis using the previously described methods. The subsequent step involved the use of synthesised cDNA samples for the purpose of conducting an immunomodulation investigation. The investigation involved the analysis of gene expression pertaining to immune response and antioxidant activity, specifically focusing on GPX-1, GST-mu, CuZnSOD, lysozyme-G (Lyso-G), apolipoprotein A-1 (ApoA-1), heat shock protein (HSP-70), and nuclear factor erythroid-related factor 2 (NRF-2), using quantitative polymerase chain reaction (qPCR). Due to the observed toxicity of the synthesised *rLr*CuZnSOD towards fish, even after undergoing multiple purification steps, it was deemed unsuitable for inclusion in this particular phase of the experimental procedure.

### 2.10. Development of Indirect ELISAs for Catalase (rLrCAT), CuZnsod (rLrCuZNSOD), GPx-1 (rLrGPX-1) and GST-mu (rLrGST-mu)

The standard curve was generated by employing purified recombinant proteins of CAT, CuZnSOD, GST-mu, and GPX-1 from the rohu species in conjunction with the corresponding antibodies produced in rabbits. In this study, various dilutions of purified r*Lr*CuZnSOD, r*Lr*GST-mu, r*Lr*GPX-1, and r*Lr*CAT were prepared using a two-fold dilution method. The dilutions ranged from 1600 ng/mL to the 11th dilution for r*Lr*CuZnSOD, 800 ng/mL to the 11th dilution for r*Lr*GST-mu and r*Lr*GPX-1, and 200 ng/mL to the 11th dilution for r*Lr*CAT. These dilutions were made in 50 µL of Tris-buffered saline (TBS). Subsequently, the diluted samples were kept overnight at 4 °C in duplicate wells of ELISA plates. The wells underwent a triple wash using TBST (Tris-buffered saline with 0.05% Tween 20). Following this, the remaining locations were subjected to a blocking procedure using a solution containing 3% bovine serum albumin (BSA) for a duration of 2 h. In addition, a volume of 100 µL of a dilution of CuZnSOD at a concentration of 500 µg/mL, 40 µg/mL of GPX-1, 1 µg/mL of GST-mu, and 5 µg/mL of catalase-specific antibody was introduced into each well for a duration of 1 h. The wells underwent a meticulous washing procedure using TBST solution. Subsequently, 50 µL of HRP-labelled secondary antibody (specifically, goat anti-rabbit antibody obtained from Genei, India) was added to the wells. This secondary antibody was diluted at a ratio of 1:5000 in TBST. The mixture was then incubated for a duration of 1 h. The wells underwent a second round of washing using TBST. Subsequently, the reaction was seen following the addition of 100 µL of TMB/H_2_O_2_ (Genei, India) to each well. Following a duration of 10 min, the reaction was terminated by the addition of 100 µL of 1 N sulphuric acid. Subsequently, the absorbance of the reaction mixture was quantified at a wavelength of 450 nm using an automated plate reader (Imax Microplate Reader, Bio-Rad). The standard curve was generated through the construction of a graph that depicted the varying amounts of CuZnSOD, GST-mu, GPX-1, and Catalase recombinant proteins in a series of dilutions (Figure S9).

### 2.11. Antioxidant Protein Concentration in L. rohita Serum during Biotic and Abiotic Stress

As detailed previously, the blood samples from *A. hydrophila* and ammonia-challenged *L. rohita* were drawn at various time points post-exposure (0, 6, 12, 24, 48 and 72 h). The separated sera were stored at −20 °C to estimate antioxidant proteins by indirect ELISA.

### 2.12. Statistical Analysis 

One-way ANOVA was used to analyse the variations in expression levels, which was followed by Duncan’s multiple range tests. A significance value of *p* < 0.05 was used to define it. The statistical analysis was performed using SPSS software, version 22.

## 3. Results

### 3.1. Cloning and Sequence Characterization of LrCAT, LrCuZnSOD, LrGPX-1 and LrGST-mu

**Figure 1:**
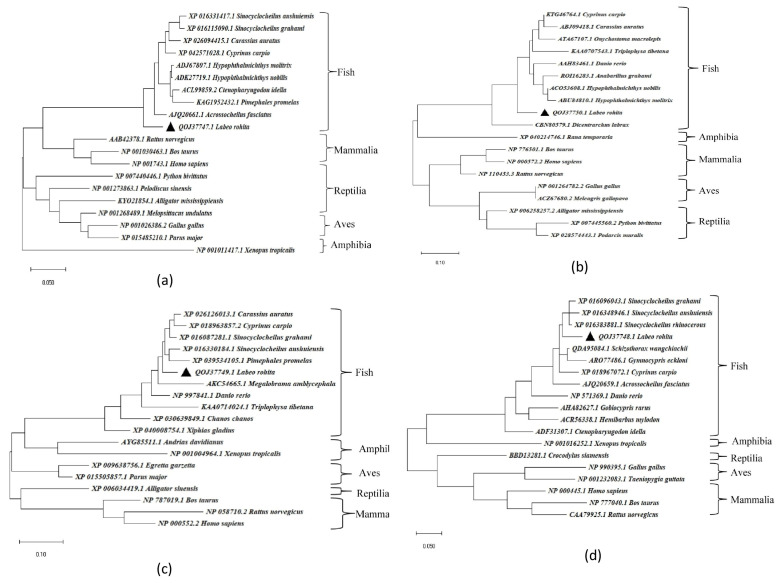
Based on the amino acid sequence alignment of *Lr*CAT (**a**), *Lr*GPX-1 (**b**), *Lr*GST-mu (**c**), and *Lr*CuZnSOD (**d**) and the evolutionary distance of the antioxidants observed in between different classes, i.e., fish, amphibia, aves, reptilia, and mammalia, the evolutionary relationship of the taxa is depicted as a tree constructed by the neighbour-joining method. ▲ represents the specific amino acid sequence of antioxidant gene found in *L. rohita*. The phylogenetic tree was inferred from evolutionary distances, and its branch lengths are shown to scale.

**Figure 2:**
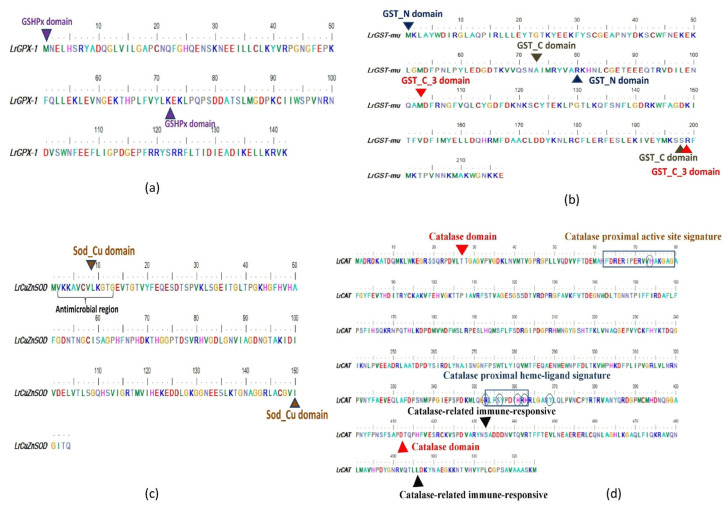
The domain architecture of *Lr*GPX-1 (**a**), *Lr*GST-mu (**b**), *Lr*CuZnSOD (**c**), and *Lr*CAT (**d**) is depicted here, with the domains denoted by ▼/▲ and the respective names appearing at the beginning and end of the sequence. Box (**d**) represents the signatures of the proximal active site and heme-ligand of the catalase. The diverse colours exclusively denote distinct amino acids.

**Figure 3:**
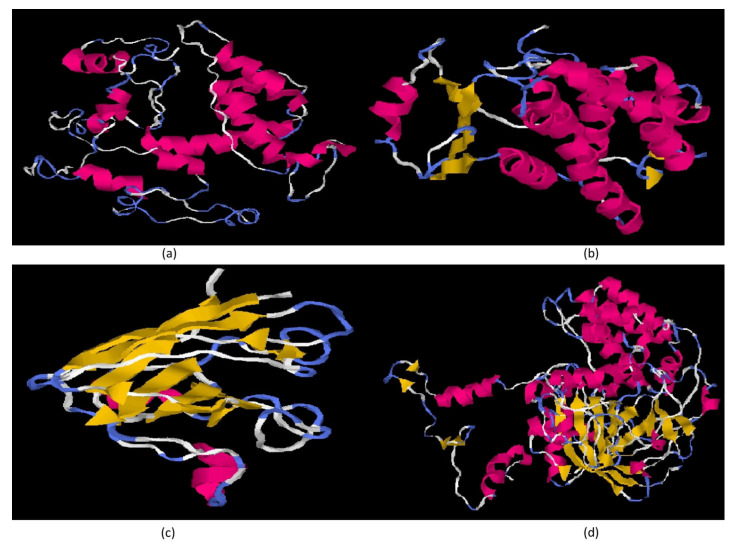
The three-dimensional structures of *L. rohita* GPX-1 (**a**), GST-mu (**b**), CuZnSOD (**c**), and CAT (**d**) were predicted, and the secondary structure elements are represented by pink ribbon-like structures for α-helices, yellow arrows for β-sheets, and the backbone is depicted in blue.

Full-length CDs of four major antioxidant genes of *L. rohita*, GPX-1, GST-mu, CuZnSOD and CAT were amplified, and the confirmed sequences were submitted to the NCBI database (accession numbers in Table 2). The phylogenetic trees of these antioxidant genes were constructed, which revealed that *L. rohita* antioxidant genes are closely related to the antioxidant genes of other fish species. Interestingly, the phylogenetic tree of fish catalase shares a common clade with mammal catalase genes (Figure 1a). However, the other three sequences, i.e., GPx-1 (Figure 1b), GST-mu (Figure 1c) and CuZnSOD (Figure 1d), showed a close relationship with amphibians. The domain architectures of the proteins were constructed and revealed the occurrence of functionally important domains in the antioxidant genes of *L. rohita* i.e., GSHPx domain (1–72 amino acids) in *Lr*GPX-1 (Figure 2a); GST_N domain (1–180 amino acids), GST_C domain (73–189 amino acids), GST_C_3 domain (103–198 amino acids) in *Lr*GST-mu (Figure 2b); Sod_Cu domain (9–150 amino acids) in *Lr*CuZnSOD (Figure 2c) and catalase domain (27–412 amino acids), catalase-related immune responsive domain (433–496 amino acids), catalase proximal active site signature domain (64FDRERIPERVVHAKGAG80) and catalase proximal heme-ligand signature domain (354RLFAYPDTH362) [17] in *Lr*CAT (Figure 2d). The 3D structure of rohu GPX-1 (Figure 3a), GST-mu (Figure 3b), CuZnSOD (Figure 3c) and CAT (Figure 3d) were predicted with secondary structure elements in pink ribbon-like structures (α-helices), yellow arrows as β-sheets, and the backbone in blue.

### 3.2. Characterization of Antimicrobial Peptide

The antimicrobial activity of a 12-mer peptide VKKAVCVLKGTG in CuZnSOD sequence (Figure 2c) was predicted using the AMPA web server (http://tcoffee.crg.cat/apps/ampa/do, accessed on 27 July 2019). [66] and commercially synthesised by Genxbio, Delhi. Various concentrations (0, 2.08, 4.16, 8.33, 16.66, and 33.3 µM) of the peptide were tested against one Gram-positive bacterium (*S. aureus*) and two Gram-negative bacteria, namely *A. hydrophila* and *Edwardsiella tarda*, to evaluate their antimicrobial activity. However, the antimicrobial peptide did not exhibit significant antibacterial activity even at a concentration of 33.3 µM, and therefore, it was not further utilised in subsequent studies.

### 3.3. Ontogeny and Tissue-Specific Expression Analysis of Antioxidant Genes

**Figure 4:**
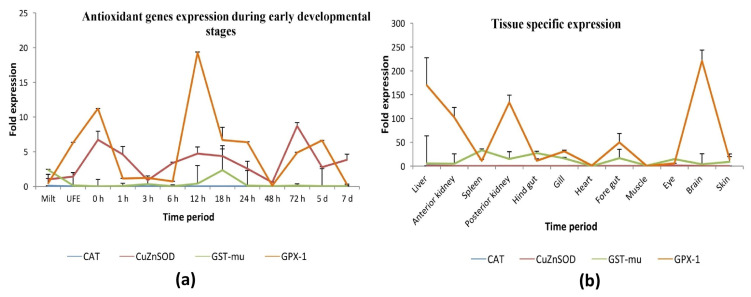
GPX-1, GST-mu, CuZnSOD, and CAT gene expression analyses at early developmental stages (**a**) and in various tissues (**b**) of *L. rohita* (*n* = 3).

During the ontogeny study, it was observed that the expression of *Lr*GPX-1 was much higher across several developmental stages in comparison to the other three antioxidant genes found in *L. rohita*. Furthermore, the highest expression of the mRNA transcript of *Lr*GPX-1 was seen at 12 h and 0 h post-fertilization. The expression of *Lr*CuZnSOD exhibited a significantly higher level at 72 h post-fertilization in comparison to the other phases of development. The expression of the *Lr*GST-mu gene was observed to be significantly elevated in the milt and in the 18 h post-fertilization stage, which corresponds to the immediate period following hatching. During the course of the ontogeny investigation, it was noted that the expression of *Lr*CAT appeared to be notably diminished. However, it was observed that the *Lr*CAT gene exhibited a high level of expression in milt in comparison to other stages of early development (Figure 4a).

Real-time polymerase chain reaction (RT-PCR) was employed to detect the expression levels of four antioxidant genes across twelve different tissues in *Labeo rohita*. The comprehensive comparative analysis conducted on the expression patterns of antioxidant genes in *L. rohita* showed that *Lr*GPX-1 exhibited a widespread distribution across all examined tissues. Conversely, the expression levels of *Lr*CAT were found to be consistently low in all tissues. It is noteworthy that the mRNA expression of *Lr*CuZnSOD was observed to be significantly elevated in the eye and liver relative to other tissue. The expression levels of the *Lr*GST-mu gene were found to be highest in the spleen and hindgut while being significantly lower in the heart tissue. Moreover, the expression of *Lr*GPX-1 was shown to be elevated in the brain, with subsequent high levels noticed in the liver, anterior kidney, and posterior kidney. The *Lr*CAT gene exhibited a dominating expression pattern in liver tissue, with only weak expression observed in the other tissues studied (Figure 4b).

### 3.4. LrCAT, LrCuZnSOD, LrGPX-1 and LrGST-mu Transcription Analysis Using Three Different Types of Pathogen Models and under Abiotic Stress

#### 3.4.1. Bacterial Infection 

**Figure 5:**
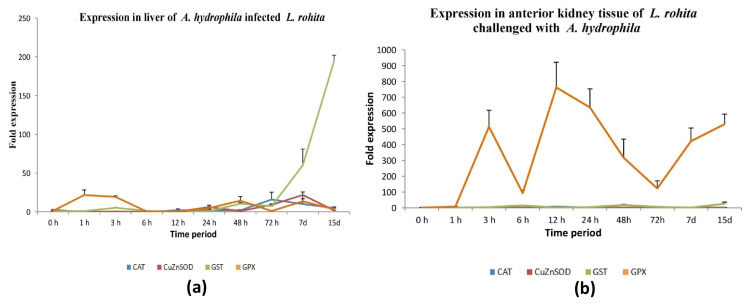
Expression of GPX-1, GST-mu, CuZnSOD, and CAT genes in *L. rohita* liver (**a**) and kidney (**b**) tissues at various time points following *Aeromonas hydrophila* challenge (h—hours following challenge; d—days following challenge; *n* = 3).

A mortality rate of 45% was observed in the experimental tanks during *A. hydrophila* challenge to rohu. The transcript levels of four antioxidant genes in liver tissue were seen to be somewhat consistent, except for GST-mu, which exhibited elevated expression during the late infection time points. The study observed a considerable up-regulation of GPx-1 expression during the initial stages of bacterial infection, specifically at 1 hpc and 3 hpc, which was subsequently followed by a down-regulation. The genes catalase, CuZnSOD, and GST-mu exhibited considerable up-regulation during the later stages of bacterial infection in liver tissue, with Catalase showing increased expression at 72 h post-infection, CuZnSOD at 24 h post-infection, and GST-mu at 7 days post-infection. Nevertheless, a notable decrease in catalase expression was noticed in the liver during the initial time intervals (Figure 5a and Figure S5).

The transcriptional analysis of four antioxidant genes in *L. rohita* showed that, in the presence of bacterial infection, the expression of GPx-1 was more pronounced in the anterior kidney tissue compared to the remaining three antioxidant genes. The GPx-1 gene exhibited a notable increase in expression at the 3 h mark following infection. In contrast, the expression levels of the remaining three antioxidant genes, namely GST-mu, catalase, and CuZnSOD, were seen to be increased at 6, 12 and 24 hpi, respectively, as depicted in Figure 5b and Figure S5.

#### 3.4.2. Parasitic Infection 

**Figure 6:**
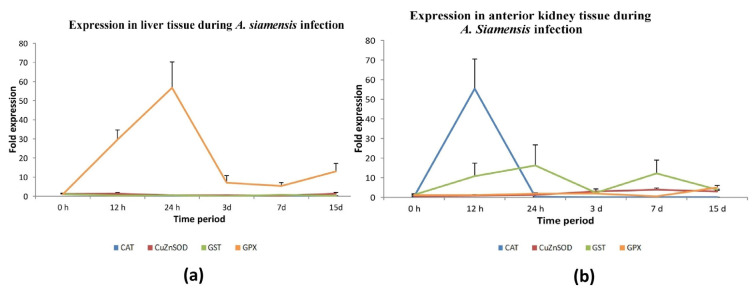
Expression of the GPX-1, GST-mu, CuZnSOD, and CAT genes in *L. rohita* liver (**a**) and anterior kidney (**b**) tissue at various points after the challenge (h—hours after the challenge; d—days after the challenge) following infection with *Argulus siamensis* (*n* = 3).

The transcript level of GPx-1 was seen to be consistently elevated compared to the other three antioxidant genes for the whole duration of the *A. siamensis* challenge in liver tissue. The expression of GPx-1 was found to be significantly increased at both 12 h and 24 h after parasite infection. In response to parasite infection, the expression of CAT and GST-mu was observed to be down-regulated. The levels of CuZnSOD expression did not show significant changes during the different time periods following the parasite infection, as depicted in Figure 6a and Figure S5.

After being exposed to *A. siamensis*, there was a notable increase in the expression levels of CAT and CuZnSOD in the anterior kidney tissue at 12 hpi. In contrast, it was shown that the expression of GPX-1 exhibited a statistically significant increase at 15 days post-infection compared to the other time periods. Nevertheless, no substantial alteration was observed in the expression of GST-mu after parasite infection in the anterior kidney tissue at any point in time (Figure 6b and Figure S5).

#### 3.4.3. Poly I:C Induction 

**Figure 7:**
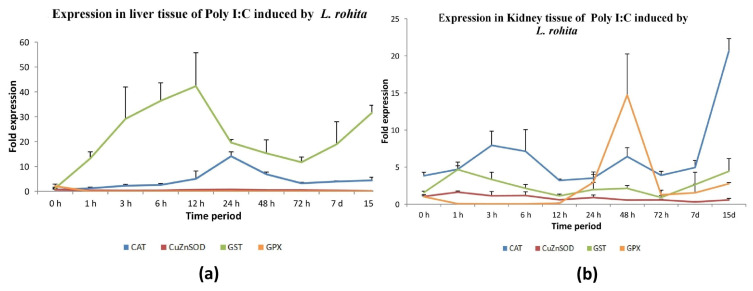
Expression of the GPX-1, GST-mu, CuZnSOD, and CAT genes in *L. rohita* liver (**a**) and anterior kidney (**b**) tissues at various time points following poly I:C induction (h—hours following induction; d—days following induction; *n* = 3).

The expression levels of GST-mu and CAT in the liver tissue of poly I:C-induced rohu were significantly elevated compared to GPX-1 and CuZnSOD. The levels of GST-mu and CAT expression exhibited a considerable increase throughout the course of the post-induction period, with the peak transcript levels seen at 12 and 24 h, respectively. In contrast, the expression of GPX-1 and CuZnSOD was notably reduced subsequent to post-induction, with the exception of a little increase in CuZnSOD expression observed at the 24 h mark following poly I:C induction (Figure 7a and Figure S5).

The expression of all four antioxidant genes in the anterior kidney tissue of rohu exhibited a paradoxical pattern when activated with poly I:C. At 48 h post-stimulation, a substantial up-regulation of GPX-1 was observed. The genes CAT, CuZnSOD, and GSTmu exhibited up-regulation over the entire stimulation period. However, it is noteworthy that all three genes had a considerable down-regulation at 12 h following poly I:C stimulation, as depicted in Figure 7b and Figure S5.

#### 3.4.4. Abiotic Stress

**Figure 8:**
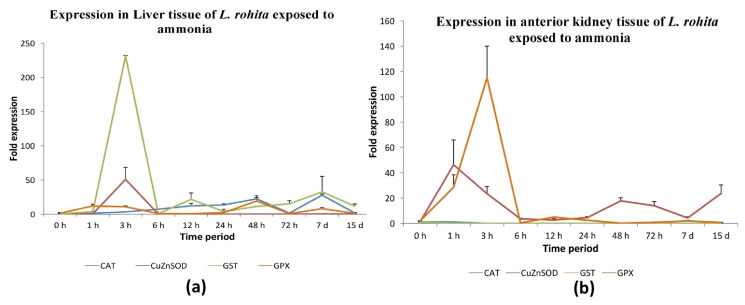
Expression of the GPX-1, GST-mu, CuZnSOD, and CAT genes in the liver (**a**) and anterior kidney (**b**) tissues of *L. rohita* at various times following ammonia exposure (h—hours following induction; d—days following induction; *n* = 3).

The expression levels of antioxidant genes, including GPX-1, GST-mu, CuZnSOD, and CAT, were seen to be considerably increased at 1, 3, and 6 h after exposure to ammonia in the liver tissue of *L. rohita*, as depicted in Figure 8a and Figure S5. In the anterior kidney tissue, it was observed that the expression of catalase and GST-mu was down-regulated, whereas the expression of CuZnSOD and GPX-1 was up-regulated at 1 h after exposure to ammonia (Figure 8b and Figure S5).

### 3.5. Recombinant Protein Production and Their Characterization

**Figure 9:**
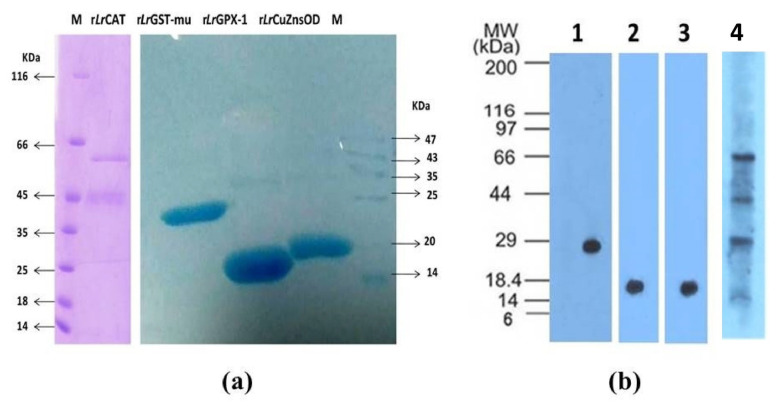
Purification of recombinant antioxidant proteins, lanes r*Lr*CAT, r*Lr*GST-mu, r*Lr*GPX-1, and r*Lr*CuZnSOD represent purified recombinant proteins of respective genes (**a**). Lane M: protein molecular weight marker. (**b**) The Western blot of the three recombinant antioxidant proteins, r*Lr*GST-mu (1), r*Lr*GPX-1 (2), r*Lr*CuZnSOD (3), and r*Lr*CAT (4).

All four recombinant proteins were expressed in the soluble fraction in the bacterial expression system. A quantity of 3–4 mg of recombinant proteins per sample was successfully purified, as depicted in Figure 9. The antibody targeting these proteins was generated in rabbits through the administration of protein at a dose of 200 µg per animal. Two further booster doses were administered on day 14 and day 21 following the initial injection. On day 30, purified antibodies were obtained from the sera of collected blood samples and utilised in an indirect enzyme-linked immunosorbent test (ELISA).

#### Antioxidant and Antimicrobial Activities

**Figure 10:**
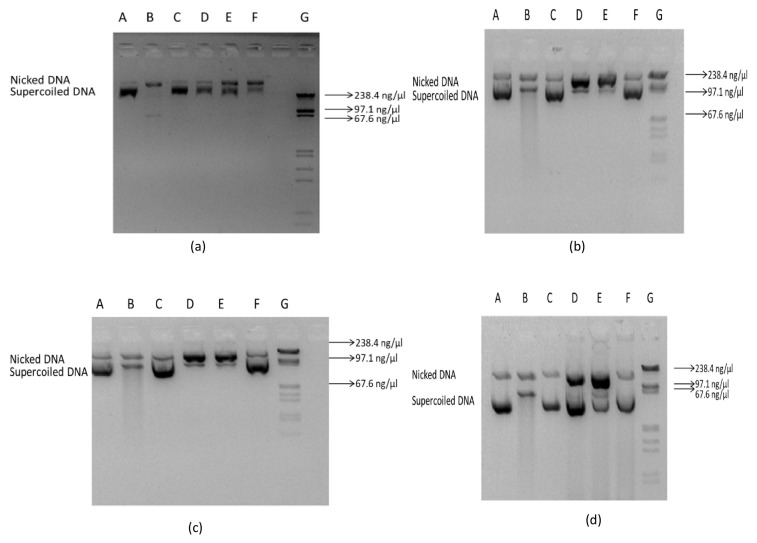
DNA damage protection assay of r*Lr*CuZnSOD (**a**), r*Lr*GST-mu (**b**), r*Lr*GPX-1 (**c**), and r*Lr*CAT (**d**). (A) pBR322 DNA without UV and H_2_O_2_; (B) pBR322 DNA + UV + H_2_O_2_; (C) pBR322 DNA + BHA + UV + H_2_O_2_; (D) pBR322 DNA + Recombinant protein 6.25 µg/mL + UV + H_2_O_2_; (E) pBR322 DNA + Recombinant protein 12.5 µg/mL + UV+H_2_O_2_; (F) pBR322 DNA + Recombinant protein 25 µg/mL + UV + H_2_O_2_; and (G) λ-marker.

The DNA protection activity of the recombinant proteins of antioxidant genes was determined. The activity seems to be higher at the concentration of 25 µg/mL of r*Lr*GPX-1, r*Lr*GST-mu, r*Lr*CuZnSOD, and r*Lr*CAT recombinant proteins (Figure 10). The minimum bactericidal concentration (MBC) of r*Lr*GPX-1, r*Lr*GST-mu, r*Lr*CuZnSOD, and r*Lr*CAT was assessed to determine their antimicrobial activity against both Gram-negative (*A. hydrophila*) and Gram-positive (*S. aureus*) bacteria. After 24 h of incubation, the MBC values for r*Lr*GPX-1, r*Lr*GST-mu, and r*Lr*CuZnSOD were observed to be 12 µM, 8 µM, and 11.5 µM, respectively. However, no antimicrobial activity was observed for r*Lr*CAT, even at 20 µM concentration.

### 3.6. Immunomodulation Study

The study investigated the comparative survival rates of fish in relation to bacterial infection caused by *A. hydrophila*, specifically focusing on the effects of r*Lr*GPX-1, r*Lr*GST-mu, and r*Lr*CAT. The results indicated that the respective survival percentages were 48%, 50%, and 40%. The degree of protection conferred by these proteins was assessed by analysing the expression patterns of various immune-related genes, including apolipoprotein-A1, lysozyme G, CuZnSOD, GPx-1, GST-mu, Hsp-70, and nuclear factor-2, in the liver and kidney tissues of rohu fish infected with the recombinant proteins at different time intervals following exposure.

ApoA1 is a major abundant plasma HDL protein in mammals and fish, having diverse protective roles such as neutralizing lipopolysaccharides, antiviral activity and inhibiting inflammatory cytokines. The expression of this gene showed a cascade of changes in all the above proteins-treated fish followed by *A. hydrophila* infection. The r*Lr*CAT treated rohu, when challenged with *A. hydrophila*, a significant up-regulation of ApoA1 transcript level was observed at 6 hpi and 12 hpi of PBS-control rohu kidney samples. In the liver of r*Lr*CAT-treated fish, the expression of ApoAI was up-regulated at 0, 12, and 24 hpi compared to their respective PBS controls. CuZnSOD is one of the important antioxidant enzymes that mediate bacterial intracellular replication. The expression of this gene did not reveal any significant change in the kidney tissue of r*Lr*CAT-treated rohu. An important intracellular antioxidant enzyme GPX-1 expression level was observed in all the targeted protein-induced samples. The transcript level was increased at 0 hpi in r*Lr*CAT-treated rohu kidney and liver samples. Furthermore, the expression of HSP-70 transcripts during different antioxidant protein exposures was observed. r*Lr*CAT-induced HSP-70 expression in the rohu kidney showed a significant up-regulation at 6 hpi. In the liver, the expression of HSP-70 was up-regulated after 12 hpi up to 48 hpi compared to the control. The expression of lyso-G in r*Lr*CAT-treated rohu kidney tissues showed a significant up-regulation at 0 and 48 hpi compared to the control. 

On the other hand, in liver tissues, the expression was down-regulated at 0 and 6 h of the bacterial challenge compared to their controls. Nuclear erythroid-related factor-2 is a ubiquitous master transcription factor that up-regulates antioxidant response elements mediated expression of antioxidant enzymes. Here, the expression of NRF-2 regulated by different recombinant antioxidant proteins was observed. The expression of NRF-2 was significantly up-regulated following r*Lr*CAT induction at 0 h post *A. hydrophila* challenge in the kidney tissue. In addition, the liver tissue of r*Lr*CAT-treated rohu displayed up-regulation of NRF-2 during all other time periods. The transcript level of GST-mu significantly increased up to 12 hpi and decreased afterwards in liver tissue; however, no significant changes in its expression were observed in kidney tissue (Figure S8). 

During r*Lr*GPX induction, the transcript level of ApoA1 increased in all of the time periods compared to their respective controls and was significantly higher at 24 and 48 hpi of bacterial challenge in kidney tissues. In liver tissues, the expression was down-regulated, and CuZnSOD expression was up-regulated in r*Lr*GPX-induced rohu kidney samples. In liver tissues, the expression of the CuZnSOD was up-regulated up to 12 hpi and down-regulated up to 48 hpi compared to the control. During the induction of r*Lr*GPX, the expression of GPX-1 was up-regulated at all the time periods of post-bacterial challenge compared to their controls. In the liver, the transcript level was high in control, at 0 and 48 hpi, respectively. The transcript level of HSP-70 was increased up to 12 hpi and decreased afterwards up to 48 hpi in r*Lr*GPX-treated rohu kidney tissue, while in the liver, the HSP-70 transcript was up-regulated at 6 hpi and down-regulated at other time periods compared to their respective controls. The transcript level of lyso-g was down-regulated in liver tissue compared to their controls, while in the kidney, lyso-g was significantly increased at 24 hpi. NRF-2 expression was significantly up-regulated in r*Lr*GPX-induced kidney samples at 0 and 24 hpi. The expression of GST-mu was observed to be significantly down-regulated in the r*Lr*GPX-induced kidney and liver tissues compared to the control. However, in liver tissue, the expression of NRF-2 was down-regulated at 0 hpi (Figure S6). 

The r*Lr*GST-treated rohu kidney samples showed a significant up-regulation in ApoAI at 48 hpi compared to the PBS-treated control group. In liver tissues, the expression of ApoA1 was rather down-regulated at all the time periods compared to their respective control groups. In the r*Lr*GST-induced rohu kidney and liver samples, a significant up-regulation of CuZnSOD was observed at all the time periods compared to their respective PBS control. Similarly, GPX-1 expression was up-regulated in the kidney tissue at all times, contrary to its down-regulation at 0, 12, and 48 hpi in the liver compared to their controls. The expression of HSP-70 was significantly up-regulated at 6 and 12 hpi in the kidney, while in liver tissue, the expression was significantly down-regulated at 0 and 48 hpi compared to their controls. The expression of lyso-g was up-regulated at all the time periods in both tissues. The expression of NRF-2 was mostly down-regulated or remained constant in both tissues. The expression of GST-mu was significantly down-regulated in kidney tissue following infection; however, no remarkable change was observed in the liver tissue (Figure S7). 

### 3.7. Development of Indirect ELISA to Measure the Level of Antioxidant Molecules in Rohu Serum

The sensitivity of indirect ELISA developed was found to be 3 ng (CuZnSOD), 1.5 ng (GPX-1), 1.5 ng (GST-mu) and 0.78 ng (catalase), respectively (Figure S9).

### 3.8. Antioxidant Levels in L. rohita Serum during Biotic and Abiotic Stress

**Figure 11:**
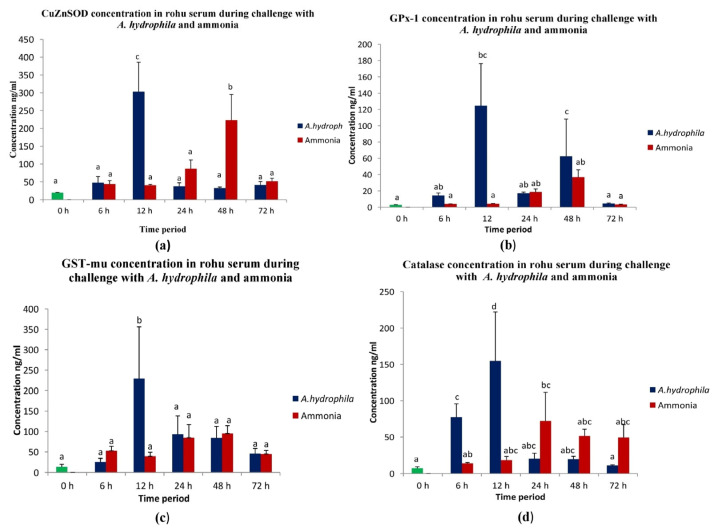
The concentration of r*Lr*CuZnSOD (**a**), rLrGPX-1 (**b**), r*Lr*GST-mu (**c**) and r*Lr*CAT (**d**) in *L. rohita* serum during challenge/induction with *A. hydrophila* and ammonia at 0 h, 6 h, 12 h, 24 h, and 48 h of post-challenge/induction. a, b, c, and d on top of the bar represent significant differences (*p* < 0.05) between naive fish (green bar) and other groups of fish at various time points.

The concentrations of antioxidant molecules during different hours of LD50/LC50 dose of *A. hydrophila* challenge/ammonia toxicity in rohu serum were measured by indirect ELISA. Interestingly, the levels of all four antioxidant molecules were found to be significantly high at 12 h of *A. hydrophila* infection, and afterwards, their levels remained low up to 72 h. However, during abiotic stress, as measured through ammonia toxicity, the concentrations of catalase and CuZnSOD were found to be significantly higher at 24 h and 48 h post-induction, respectively (Figure 11).

## 4. Discussion

Extensive research has been conducted on antioxidant enzymes in fish due to their pivotal role in safeguarding fish cells from oxidative stress and preserving their overall well-being. The significance of antioxidant enzymes in fish extends beyond the realm of fish biology, encompassing implications for both environmental and human health. Fish are frequently employed as bioindicators to assess water quality and pollution levels. Monitoring variations in the activity of antioxidant enzymes in fish can offer valuable insights into the effects of pollutants on aquatic ecosystems. The multifunctional role of antioxidants in several fish species has been extensively investigated [14,39,80,81,82,83]. However, there is limited information on most of the antioxidant genes at the sequence level, including their well-characterized functional roles in several aquaculture-important food fish species, including rohu, an Indian major carp species. Hence, it was crucial to first characterise these genes in rohu through cloning and sequencing in order to better understand their functional relevance. Hence, the current study focused on conducting a molecular and functional characterization of four significant antioxidant enzymes, namely catalase (CAT), glutathione peroxidase-1 (GPX-1), glutathione S-transferase-mu (GST-mu), and copper-zinc superoxide dismutase (CuZnSOD) in rohu, *L. rohita*. 

Further, the findings of the study advanced our knowledge of antibacterial, antioxidant and immunomodulatory roles of the recombinant proteins of these antioxidant molecules in rohu besides the development and utilization of ELISA assay systems to establish their levels under biotic and abiotic stress conditions. 

The *Lr*CAT sequence, which was analysed, comprised 525 amino acids and had an estimated molecular mass of approximately 59.57 kDa. It falls within the typical size range of 460–590 amino acids and has a molecular mass of 50–60 kDa, as commonly observed in both prokaryotes and eukaryotes [5,16,84]. Through the multiple sequence alignment, it was observed that the catalase enzyme of *L. rohita* exhibited the highest percentage of similarity when compared to *Cyprinus carpio, Hypophthalmichthys molitrix*, and *Hypophthalmichthys nobilis*. The presence of various unique domains in *Lr*CAT, such as the catalase domain, catalase immune response domain, catalase proximal active site signature domain, and catalase proximal heme-ligand signature domain, is evident from the analysis of its domain architecture and multiple alignments (Figure 1a). The last two domains of *Lr*CAT exhibited a significant degree of conservation when compared to the catalases of other vertebrates. Recent research has demonstrated that the presence of a charge-relay network is potentially necessary for the catalytic and peroxidase activities of catalases. This network stabilises the reaction intermediates and facilitates the cleavage of peroxide molecules. The process described above has been hypothesized to regulate the function of catalase by reducing the positive charge of the porphyrin radical and creating an electron-deficient oxyferryl moiety at Tyr 358 [85]. In this present investigation, a detailed examination of the *Lr*CAT sequence revealed seven key amino acid residues (His75, Asp141, Arg354, Tyr358, His362, His364, and Tyr370) that are crucial for maintaining the integrity of the electronic circuit essential for optimal enzyme performance. These residues are implicated in the intricate processes that govern the catalytic and peroxidase activities of *Lr*CAT. The clustering arrangement seen in the phylogenetic tree provides support for the shared ancestral origin of *Lr*CAT among vertebrates. This study not only enhances our understanding of *Lr*CAT domain architecture but also contributes valuable insights into the broader evolutionary context in vertebrates. 

CuZn superoxide dismutase (SOD) is a metalloenzyme with anti-oxidative properties that is widely distributed in many tissues and cell types and is present in a wide range of species [39]. The 154 amino acid sequence of *Lr*CuZnSOD that was acquired exhibited a significant degree of conservation when compared to the sequences of other teleosts, as reported by Li et al. (2010) [43] and Zhang et al. (2011) [44]. According to Zhang et al. (2019) [86], the CuZnSOD enzyme can be classified into two isoforms based on its location. The extracellular isoform of CuZnSOD possesses an N-terminal signal cleavage peptide, while the intracellular isoform lacks a signal peptide. Similar to other teleosts, the *Lr*CuZnSOD is classified as a member of the intracellular CuZnSOD family lacking signal peptide sequence. The domain architecture of *Lr*CuZnSOD includes the SOD_Cu domain, which spans from Leu9 to Ile150 amino acids. A comparable domain has also been identified in the mussel *Mytilus galloprovincialis*, spanning from Lys10 to Ile174, as described by Wang et al. in 2013 [56]. The application of multiple sequence alignment to the CuZnSOD sequence of *L. rohita*, along with other homologous sequences, has identified three sections that exhibit a high degree of conservation. These regions include the N-terminal region, the C-terminal region, and the intermediate region. Furthermore, the analysis of the evolutionary tree of *Lr*CuZnSOD in conjunction with other vertebrates demonstrates its significant similarity with several fish species. 

The expression of glutathione peroxidase has emerged as a valuable research tool for assessing environmental pollution levels and evaluating the extent of stress in fish species [87]. The involvement of GPX in the process of detoxification and the removal of hydrogen peroxide from cells through glutathione oxidation has been extensively studied [88]. The present investigation involved the isolation of GPX cDNA from the liver tissue of the rohu fish species. The *Lr*GPX-1 gene sequence consists of an open reading frame (ORF) spanning 429 base pairs, which is responsible for encoding a protein consisting of 142 amino acids. Choi et al. (2007) [81] discovered a protein of comparable size in *Carassius auratus*. The protein’s molecular weight was determined to be 16.6 kDa, while its isoelectric point was measured to be 5.6. The amino acid sequence of *Lr*GPX-1 has been determined, and it includes a signature motif known as motif 2 (LGAPCNQF). This motif is consistent with the sequences found in various species of the Cyprinidae family, such as *Acrossocheilus fasciatus*, *Carassius auratu*s, *Hypophthalmichthys nobilis*, *Ctenopharyngodon idella*, *Hypophthalmichthys molitrix*, and *Danio rerio* GPX-1 [89]. In contrast, it was shown that the conserved active site motif (WNFEKF) of *Lr*GPX-1 exhibited a single amino acid mutation, specifically the substitution of lysine with glutamic acid. Moreover, the amino acid sequences of *Lr*GPX-1 were subjected to multiple alignments, and the resulting phylogenetic tree demonstrated a shared clade with other fish species, such as *H. molitrix*, *H. nobilis,* and *Anabarilius graham*. 

The GSTs, or glutathione S-transferases, are a diverse group of enzymes involved in phase II detoxification of xenobiotics [90]. A comprehensive categorization of cytosolic GSTs has been established, encompassing more than 14 distinct classes (such as alpha, beta, delta, mu, omega, pi, sigma, tau, theta, and zeta, among others). These classes have been determined through the analysis of various factors, including protein folds, the composition of catalytic residues, thermodynamic and kinetic characteristics, specificity towards substrates, cross-reactivity with antibodies, the specific reactions they catalyse, and their susceptibility to inhibitors [91]. The present study focuses on the cloning, sequencing, and characterization of GSTmu, a specific class of GST that has been identified as having significant involvement in antioxidant defence mechanisms. Previous research has indicated that the expression of GSTmu is influenced not only by exposure to environmental pollutants but also by infectious pathogens [58]. The GSTmu contains an ORF of 654 bp, which encodes a protein of 217 aa with a molecular weight of 25.75 kDa and an isoelectric point of 5.87. The *Lr*GSTmu have no signal peptide suggesting it is cytosolic GST like *Macrobrachium rosenbergii* GST proteins. Domain architecture of *Lr*GSTmu revealed the presence of GST_N domain (1–80 aa), GST_C domain (73–189 aa) and GST_C_3 domain (103–198 aa) similar to other teleosts [58,92]. Multiple amino acid sequence alignment revealed that GST_N and GST_C domains are conserved among species. The phylogenetic analysis revealed that *Lr*GSTmu shares the same clade with *Pimephales promelas* and *Megalobrama amblycephala.* Hence, these four antioxidant genes studied here closely resemble other fish species.

To study the tissue distribution and role of these antioxidant genes in rohu, further investigation focused on examining the expression profile of antioxidant genes in particular tissues. The findings revealed that these proteins were present in all 12 tissues listed but with varying levels of abundance. The qRTPCR investigation revealed that the expression of *Lr*CAT was much higher in liver tissue compared to the other 11 tissues. The liver, being the primary organ responsible for several oxidative reactions and antioxidant defence mechanisms, often experiences elevated levels of oxidative stress resulting from excessive synthesis of reactive oxygen species (ROS). These functions could potentially contribute to the elevated expression of catalase in the liver. Consistent with our findings, previous studies have reported that catalase is mostly expressed in the liver of several fish species, including *Sebastes schlegeli* [14], *Takifugu obscurus* [93], *C. auratus* [94], and *Onychostoma macrolepis* [15]. The study revealed that CuZnSOD had a significant presence in the eye tissue of *L. rohita*, with a subsequent distribution observed in the liver tissue. The expression of CuZnSOD in many tissues of aquaculture species demonstrates the intricate nature of the process involved in eliminating reactive oxygen species (ROS). The expression of CuZnSOD was observed to be significantly elevated in the intestine and coelomocytes of *Apostichopus japonicus* [39] and in the hepatopancreas and gills of *Anodonta woodiana* [41]. Similarly, high levels of CuZnSOD expression were detected in the gill filaments and haemocytes of *Chlamys farreri* [43], as well as in the gills and haemocytes of *Pinctada fucata* [95]. Furthermore, CuZnSOD expression was found to be prominent in the liver and spleen of *H. molitrix* [44].

In the aforementioned research, it was observed that the expression of CuZnSOD was present in tissues that are involved in the removal of reactive oxygen species (ROS) as well as other immunologically significant functions. Previous research has indicated the significance of the eye, as the ocular surface is consistently exposed to potentially harmful materials such as bacteria and toxic compounds in the environment, which increases the susceptibility of the ocular surface to immunological events [96]. In a manner akin to *H. molitrix*, the liver of *L. rohita* exhibited elevated levels of CuZnSOD expression. This observation further underscores the liver’s significance in mitigating oxidative stress and maintaining equilibrium, akin to the role played by catalase. Glutathione peroxidase is a widely distributed antioxidant enzyme and was found to express highly in the brain > liver > posterior kidney > anterior kidney and also expressed moderately in all other tissues of *L. rohita.* Earlier studies showed the expression of GPX being extensively distributed in a wide range of tissues, depicting its antioxidant potential. In the species *C. auratus*, the expression of GPX was found in the liver and kidney; however, brain and intestine tissue had no expression [81]. GPX1 is abundantly present in the cytosol and mitochondria of erythrocytes, kidneys, and liver of mammals [97,98]. GPX activity has been detected in the hepatopancreas and gills of the giant freshwater prawn, *M. rosenbergii* [99], and has also been demonstrated to be present in haemocytes of the white shrimp, *Litopenaeus vannamei* [29], digestive tract of abalone, digestive gland and gills of *Unio tumidus* [100] and *Dreissena polymorpha* [101]. The presence of *Lr*GPX in the rohu brain is noteworthy and might have functional significance that needs to be investigated further. Previous investigations have found several factors that contribute to considerable differences in the expression of GSTs across various tissues.

Several factors can influence the regulation and expression of GSTs, such as the sex of the organism, developmental stage, tissue-specific factors, and the precise type of xenobiotics in contact [102]. Similarly, in our observation, the expression of GSTmu was detected in all 12 tissues of rohu with different transcript levels. The highest expression of GST-mu was observed in the spleen tissue of rohu. The expression of mu-type GST from *Haliotis discus discus* was observed in various tissues, including the gills, mantle, gonad, foot, and digestive tract [58,103]. Similarly, a Pi-type GST from the Antarctic bivalve *Laternula elliptica* exhibited widespread expression in tissues such as gonads, digestive glands, mantle, gills, and intestines [104]. Another widely expressed gene, a mu-type GST from the rock shell *Thais clavigera*, showed the highest expression level in the gills [105]. In *M. rosenbergii*, the expression of the GST mu was also recorded for wide distribution in all the tissues [58]. The wide distribution and tissue-specific expression pattern of GST isoforms has also been recorded in various aquatic species [54,106,107]. 

The expression of antioxidant genes during the developmental stages has not been widely studied in fish. Here, we looked into the expression pattern of four antioxidant genes in the developmental stages of *L. rohita and* milt collected from rohu males. The expression of catalase and GST-mu was observed to be very high in milt, which signifies the importance of these antioxidants in protecting spermatozoa against oxidative damage. Earlier studies revealed that catalase has been shown to play an important role in male fertility by balancing oxidative stress, which may cause damage to sperm, thereby reducing fertility [108]. In our previous study, we also noticed a high level of expression of the antioxidant NKEF-B in milt [66]. Currently, our study recorded a paradoxical way of expression of antioxidants in the developmental stages of rohu, possibly implying the phenomenon of adaptation to ROS-induced natural oxidative stress.

To understand the role of these antioxidant genes during biotic and abiotic stresses, an expression analysis of these genes was undertaken that revealed their potential role like other living beings. A wide-ranging expression change of antioxidant genes was observed in different tissues during different biotic (microbial invasion) and abiotic stress in various fish species to counteract the ROS. In our study, the changes in expression of the *Lr*CAT, *Lr*GPX-1, *Lr*GST-mu and *Lr*CuZnSOD were observed in liver and anterior kidney tissues during *A. hydrophila*, *A. siamensis*, poly I:C induction (as biotic stress factors) and ammonia challenge (as abiotic stress factor). The liver and anterior kidney tissues were used here as the target tissues to observe antioxidant transcription, as these organs play a major role during stress conditions. All through microbial infection and toxic chemical interaction in fish species, measuring free radical production and antioxidant levels are crucial steps of the host immune defence system [2]. Here, in our study, a varied transcript level of antioxidant genes was observed during the above-mentioned challenge study in different periods, indicating the defensive role of antioxidants in the ROS produced due to oxidative stress. These observed alterations in antioxidant gene expression patterns resonate with findings from prior infection studies, emphasizing the conserved nature of the host’s response to stress [14,17,29,46,54,81,109,110]. Importantly, our study contributes further evidence by revealing similar expression changes across various tissues in response to different stressors, reinforcing the critical role of antioxidant defence in fish facing diverse challenges. 

To evaluate the exact functional role of these rohu antioxidant molecules, we expressed the recombinant proteins from the cloned genes in the bacterial system. Cellular DNA is vulnerable to oxidative damage caused by OH-free radicals, well known for their deleterious effects. The UV-photolysis of H_2_O_2_ generates OH radicals, contributing to oxidative damage to most proteins and DNA. The purified recombinant proteins of *Lr*CAT, *Lr*GPX-1, *Lr*GST-mu, and *Lr*CuZnSOD exhibited notable in vitro antioxidant activities, effectively shielding DNA from UV-induced damage at variable concentrations. This aligns with findings from other studies, where antioxidant enzymes from various species have been reported to safeguard supercoiled DNA against oxidative stress [111,112]. In our study, we explored further the antibacterial properties of recombinant antioxidant enzymes, typically recognized for their antioxidative benefits. Antioxidants are known as natural antibiotics because of their low toxicity and low risk of developing bacterial resistance [113]. Surprisingly, all tested enzymes exhibited significant antimicrobial effects against both Gram-positive and Gram-negative bacteria. This finding is quite interesting, revealing the additional role played by the antioxidant molecules in having bactericidal properties. However, the detailed mechanistic action of these molecules in causing bacteriolysis needs further investigation besides exposing their potential role in serving as an alternative to antibiotics [113]. 

The purified antioxidant proteins of *L. rohita* were further investigated in vivo to evaluate their protection abilities against bacterial (*A. hydrophila*) infection and modulate the expression level of different antioxidant, antimicrobial, immune-related and stress-related genes in the *A. hydrophila*-challenged *L. rohita*. Antioxidant genes viz., CuZnSOD, GPX-1, GST-mu play an important role in the antioxidant defence system that destroys free radicals by catalysis and maintains the redox balance of the immune system [29,54,58,63,109,110,114]. The down-regulation of these genes in GPX-1 and GST-mu treated bacteria challenged *L. rohita,* suggesting that the external supply of these proteins might influence the endogenous synthesis of CuZnSOD, GPX-1 and GST-mu transcripts during the infection process. However, in catalase-induced tissue, the up-regulation of antioxidant genes indicated that the external supply of *Lr*CAT has a less direct antimicrobial role; rather, its action is through modulation of a cascade of antioxidant molecules, as noticed here. The Nuclear factor E-2 related gene (Nrf2) is a crucial transcription factor responsible for regulating the expression of many detoxifying and antioxidant defence genes within the liver [115]. The up-regulated Nrf2 gene in the liver tissue of GPX-1, GST-mu and CAT-treated rohu indicated the activation of Nrf2 genes in response to oxidative stress and induced the expression of its target genes by binding to the antioxidant response element (ARE) [115]. The antimicrobial peptide known as apolipoprotein A-I (ApoA-I) is a very prevalent and versatile high-density lipoprotein (HDL) that plays a significant role in lipid transportation and exhibits strong antibacterial properties against a diverse array of microorganisms [72]. The decrease in the ApoA1 transcript in the initial phase and subsequent increase in the transcript level in GPX-1, GST-mu and CAT-treated rohu were also reported previously [72] in the liver following the *A. hydrophila* challenge in rohu. It might be due to acute septicemia, particularly damaged liver tissue in fish at an early stage of infection, and the subsequent increase in expression levels can be associated with repair activity of liver tissue in the survivors of this infection at a later stage [116]. According to Mohapatra et al. [61], lysozyme is a significant secretory innate immunity component that exhibits antibacterial properties against many bacteria and viruses. The observed variability in the up- and down-regulation of lyso-g expression in bacteria-infected rohu treated with purified antioxidant proteins may suggest variations in the bacterial load at specific time points or the presence of bacterial toxins that induce inflammation, thereby influencing the observed pattern.

Further investigation is required to gain a comprehensive understanding of this phenomenon [61]. The activation of the host immune system during infection is facilitated by stress-related genes known as heat shock protein (Hsp) genes [60]. In earlier studies, it was documented that the upregulation of the hsp-70 gene occurred in various fish species, including coho salmon, rainbow trout, and sea bream, in response to bacterial infection [117,118,119]. The present study observed a notable increase in the expression of the hsp-70 gene in the liver and kidney tissues of *L. rohita* triggered by GPX-1, GST-mu, and CAT. This finding indicates the potential involvement of the hsp-70 gene in the immunological response to bacterial infection in fish. 

The investigation into the functional significance of antioxidants in rohu at the protein level in blood was deepened by implementing an indirect ELISA technique. This innovative approach sheds light on the elevated levels of antioxidant molecules detected in the blood during biotic stress induced by *A. hydrophila* and abiotic stress triggered by ammonia at various time points after infection/induction. This intriguing observation strongly suggests the potential immunomodulatory and protective roles of antioxidants in response to stressful conditions. Notably, the concentration of these molecules was shown to be elevated during the initial stage of bacterial infection, suggesting their potential protective function in mitigating bacteria-induced stress in fish. Conversely, during ammonia-induced stress, the concentration of these molecules was found to be higher during a later stage. This intriguing observation strongly suggests the potential immunomodulatory and protective roles of antioxidants in response to stressful conditions. 

Further, the developed assay systems could be potentially biomarkers in various stressful events in carp. 

## 5. Conclusions

In the present study, the four most important antioxidants (CAT, CuZnSOD, GPX-1, GST-mu) of *L. rohita* were cloned and characterized in detail for the first time. The antioxidants were found to be widely expressed in different tissues and developmental stages of *L. rohita.* The expression of the antioxidants also showed significant changes in three infection models in both liver and anterior kidney tissues. The purified recombinant proteins of the above antioxidants were produced in the bacterial expression system and functionally characterized. The purified recombinant antioxidant molecules of *L. rohita* were shown to have potent antioxidant and antimicrobial activities. From four antioxidants, three recombinant proteins, i.e., CAT, GPX-1, GST-mu, were studied for the relative percentage survival of *L. rohita* against bacterial infection; however, r*Lr*CuZnSOD was found toxic to fish that needs further investigation. The immunomodulatory role of these antioxidant molecules and their concentrations were studied by indirect ELISA during bacterial infection and ammonia induction, and the study, in total, indicated the potential role of these molecules in rohu to combat infection or reduce abiotic stress. These antioxidants could be potentially targeted as an alternative to antibiotics or immunostimulants in aquaculture.

## Figures and Tables

**Table 1:** Details of primers used in this study and their optimum annealing temperatures.

Target Gene	Primer Sequence 5′-3′	Size of PCR Amplicon (bp)	Optimum Annealing Tempera-Ture (°C)	Reference or Accession No. of Target Gene
RoCATCDs	F-CATGGCAGACAGAGATAAG R-TCACATCTTAGAAGCTGCA	1.2 kb	52	MN190714
RoCuZnSODCDs	F-CATGGTGAAGAAGGCTGTT R-TCATCAGTGGGCTAAGTGC	536	56	MN190715
RoGPX-1CDs	F-TTCGGAGTGCGTAGTAAAC R-GCTTATTTCACCCTCTTCAG	610	56	MN190717
RoGSTmu CDs	F-ATGAAATTGGCTTACTGGGA R-GTTTCACTCCTTCTTGTTTCC	657	56	MN190716
RoCATRT	F-ACCTCTACAACGCCATCT R-ATTCCACTTCCAGTTCTCAG	190	56	MN190714
RoCuZNSODRT	F-ACGGTGGACCAACTGATA R-CAAGTCATCCTCCTTCTCAT	167	56	MN190715
RoGPX-1RT	F-AGGAGAACAGCAAGAATGAA R-CAATGTCGATGGTGAGGAA	313	56	MN190717
RoGSTmuRT	F-GAAGAAGAGCAGACGAGAG R-TGTCACCAAGGAAGTTAGAG	160	52	MN190716
β-actin	F-TTGGCAATGAGAAGGTTCAGGT R-TTGGCATACAGGTCCTTACGG	139	56	[70]
GAPDH	F-AACTCACCAAGTTTTGCGACAG R-AGGTGGGAACAGGAATGCTAAG	145	56	[71]
Nrf2	F-CTGTCAGGTTCTCAGGATTG R-CACGATATGATCCAGCTTTC	410	54	Self designed
HSP70	F-CTACTCGGACAATCAGCC R-GGAATGCCAATCAACTCA	105	54	[60]
APOA1	F-TGGAGGCTGTGCGTGTA R-GCTCGCCCAGTTCATTC	164	59	[72]
LysoG	F-AAAGCAAATTCCCTCGTCGTG R-GGTTCTGGCATCGATATTCT	230	54	[61]

**Table 2:** Detail of sequence information of antioxidant genes.

Antioxidant Genes	NCBI Accession Number	Complete ORF (in Base Pair)	Encoded Protein (in Amino Acids)	Molecular Weight of the Protein (in KDa)	Isoelectric Point (pI)	SignalP (Signal Peptide)
*Lr*GPX-1	MN190717	429	142	16.46	5.66	No
*Lr*GST-mu	MN190716	654	217	25.75	5.87	No
*Lr*CuZnSOD	MN190715	465	154	15.92	5.79	No
*Lr*CAT	MN190714	525	525	59.57	6.53	No

## Data Availability

All of the data is contained within the article and the Supplementary Materials.

## Supplementary Materials

The following supporting information can be downloaded at https://www.mdpi.com/article/10.3390/antiox13010018/s1, Figure S1. Alignment of *Lr*CAT’s amino acid sequences with those of other species.; Figure S2. Alignment of *Lr*CuZnSOD’s amino acid sequences with those of other species.; Figure S3. Alignment of *Lr*GPX-1’s amino acid sequences with those of other species.; Figure S4. Alignment of *Lr*GST mu’s amino acid sequences with those of other species.; Figure S5. Expression of GPX-1, GST-mu, CuZnSOD, and CAT genes in *L. rohita’s* liver and kidney tissue during biotic and abiotic stress conditions, i.e., *A. hydrophila (a & b)*, *A. siamensis (c &d)*, *poly I:C (e & F)* and ammonia (g & h) infection/induction at different time periods (*n* = 3). The bars show the mean ± S.E. of three fish.; Figure S6. Variation in expression pattern of different immune-related and antioxidant genes, ApoA1 in kidney (a) and liver (b), Lyso-G in kidney (c) and liver (d), CuZnSOD in kidney (e) and liver (f), GPX-1 in kidney (g) and liver (h), GST-mu in kidney (i) and liver (j), nrf-2 in kidney (k) and liver (l) and hsp-70 in kidney (m) and liver of different groups of *L. rohita* (treated with r*Lr*GPX-1) at different time periods (*n* = 3). The bars show the mean ± S.E. of three fish.; Figure S7. Variation in expression pattern of different immune-related and antioxidant genes, ApoA1 in kidney (a) and liver (b), Lyso-G in kidney (c) and liver (d), CuZnSOD in kidney (e) and liver (f), GPX-1 in kidney (g) and liver (h), GST-mu in kidney (i) and liver (j), nrf-2 in kidney (k) and liver (l) and hsp-70 in kidney (m) and liver of different groups of *L. rohita* (treated with r*Lr*GST-mu) at different time periods (*n* = 3). The bars show the mean ± S.E. of three fish.; Figure S8. Variation in expression pattern of different immune-related and antioxidant genes, ApoA1 in kidney (a) and liver (b), Lyso-G in kidney (c) and liver (d), CuZnSOD in kidney (e) and liver (f), GPX-1 in kidney (g) and liver (h), GST-mu in kidney (i) and liver (j), nrf-2 in kidney (k) and liver (l) and hsp-70 in kidney (m) and liver of different groups *of L. rohita* (treated with r*Lr*CAT) at different time periods (*n* = 3). Bars represent mean ± S.E of three fish.; Figure S9. Standard curve used for quantification of normal serum CuZnSOD, GPX-1, GST-mu and CAT level in *L. rohita*.
