# Supplementary material for: Antioxidant Defence in Labeo rohita to Biotic and Abiotic Stress: Insight from mRNA Expression, Molecular Characterization and Recombinant Protein-Based ELISA of Catalase, Glutathione Peroxidase, CuZn Superoxide Dismutase, and Glutathione S-Transferase
Source: Antioxidants (Basel). 2023 Dec 21;13(1):18. doi: 10.3390/antiox13010018 (PMC10812468; doi:10.3390/antiox13010018)
Supplement: Supplementary file 1 [file antioxidants-13-00018-s001.zip › antioxidants-2674377-supplementary.pdf]

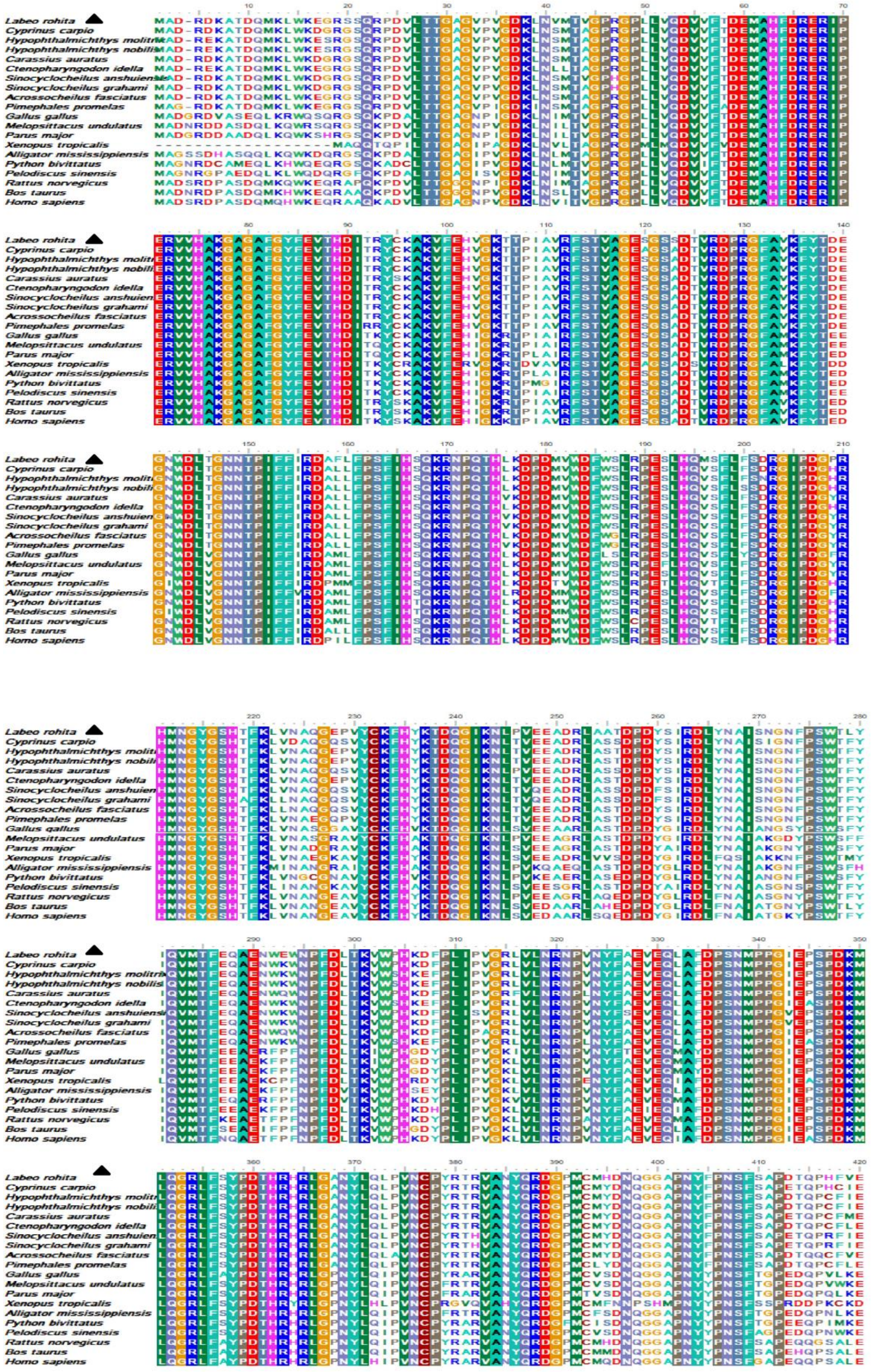
Labeo rohita
Cyprinus carpio
Hypophthalmichthys molitrix
Hypophthalmichthys nobilis
Carassius auratus
Ctenopharyngodon idella
Sinocyclocheilus anshuiensis
Sinocyclocheilus grahami
Acrossocheilus fasciatus
Pimephales promelas
Gallus gallus
Melopsittacus undulatus
Parus major
Xenopus tropicalis
Alligator mississippiensis
Python bivittatus
Pelodiscus sinensis
Rattus norvegicus
Bos taurus
Homo sapiens

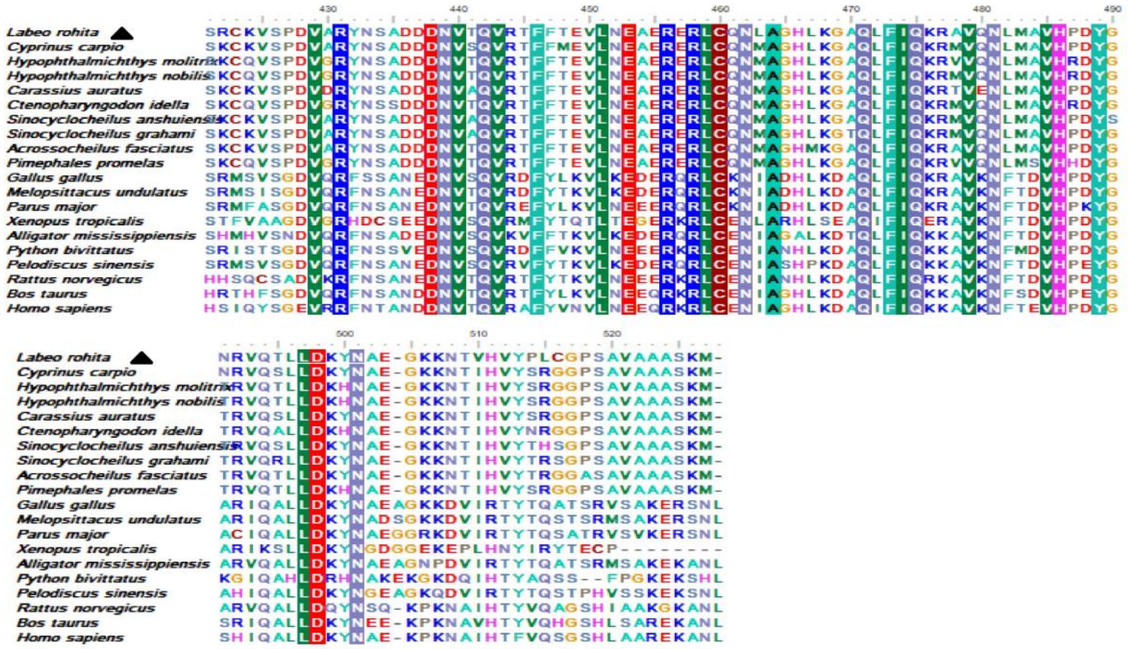

Figure S1. Alignment of *Lr*CAT's amino acid sequences with those of other species.

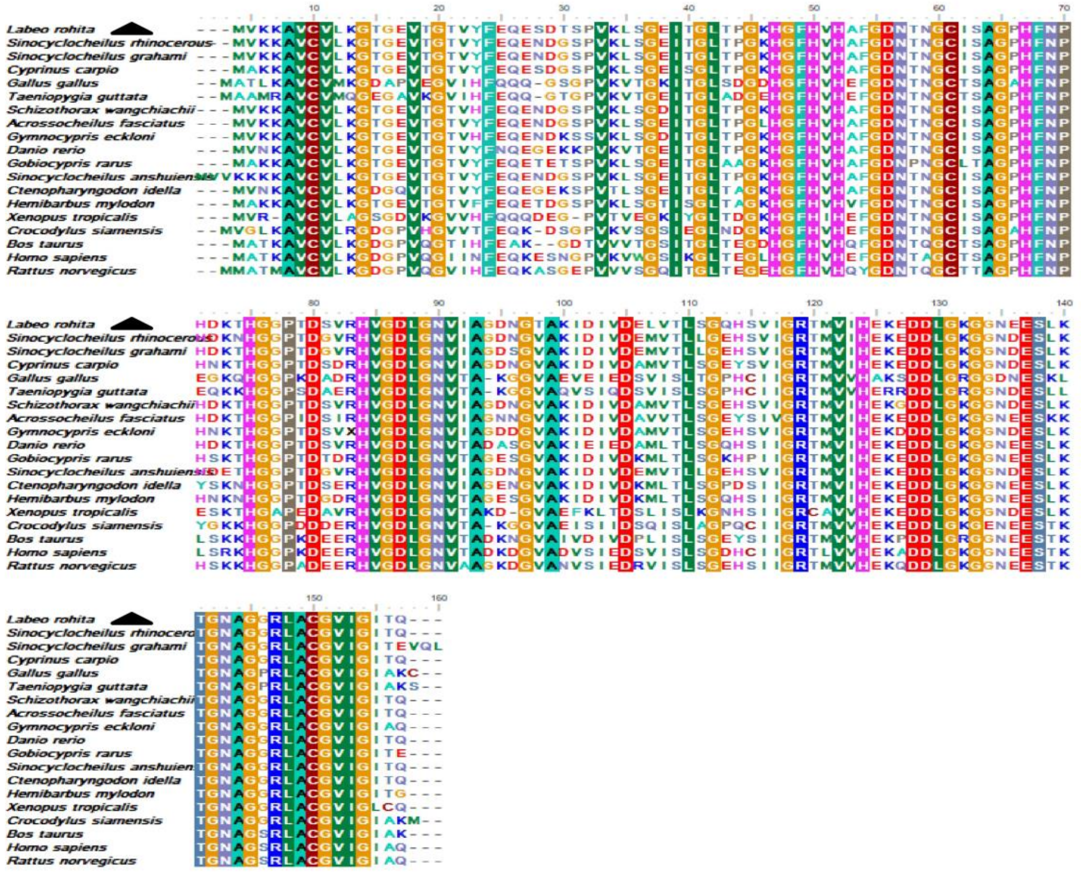

Figure S2. Alignment of *Lr*CuZnSOD's amino acid sequences with those of other species.

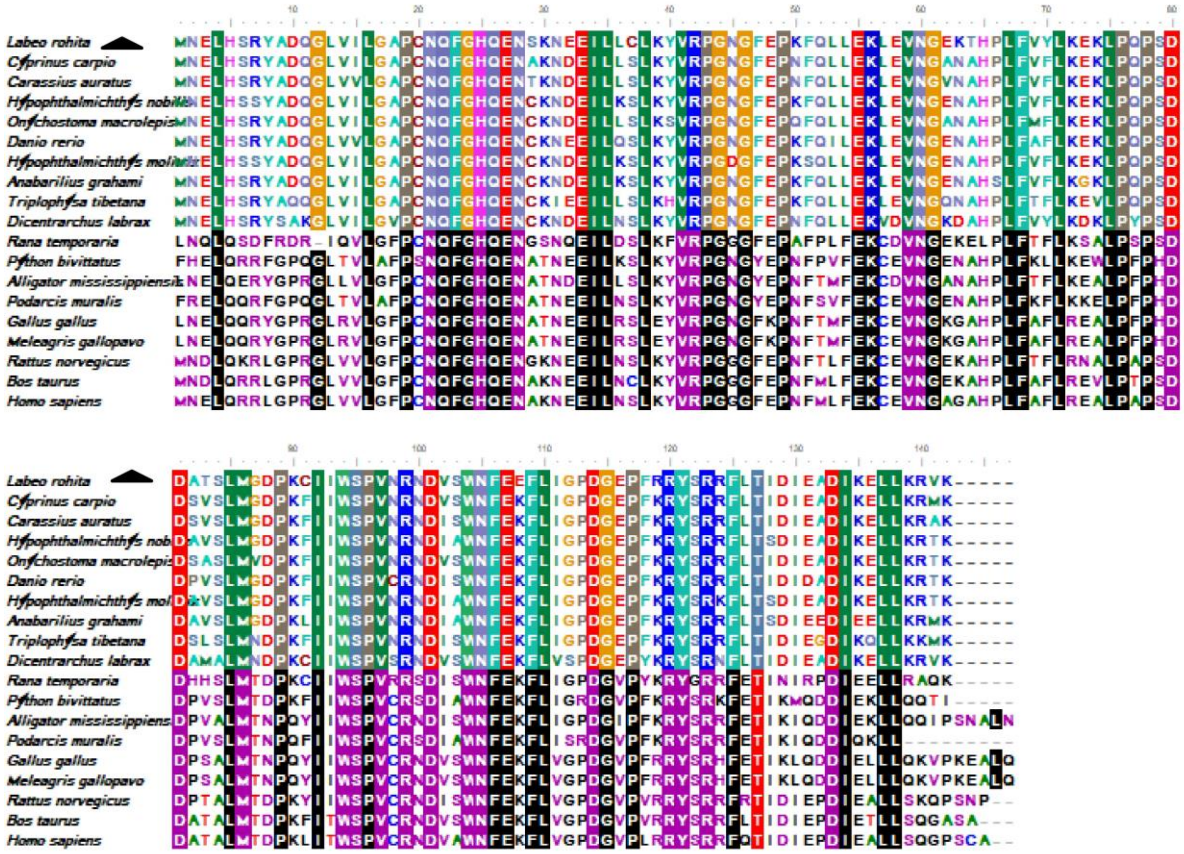

Figure S3. Alignment of *Lr*GPX-1's amino acid sequences with those of other species.

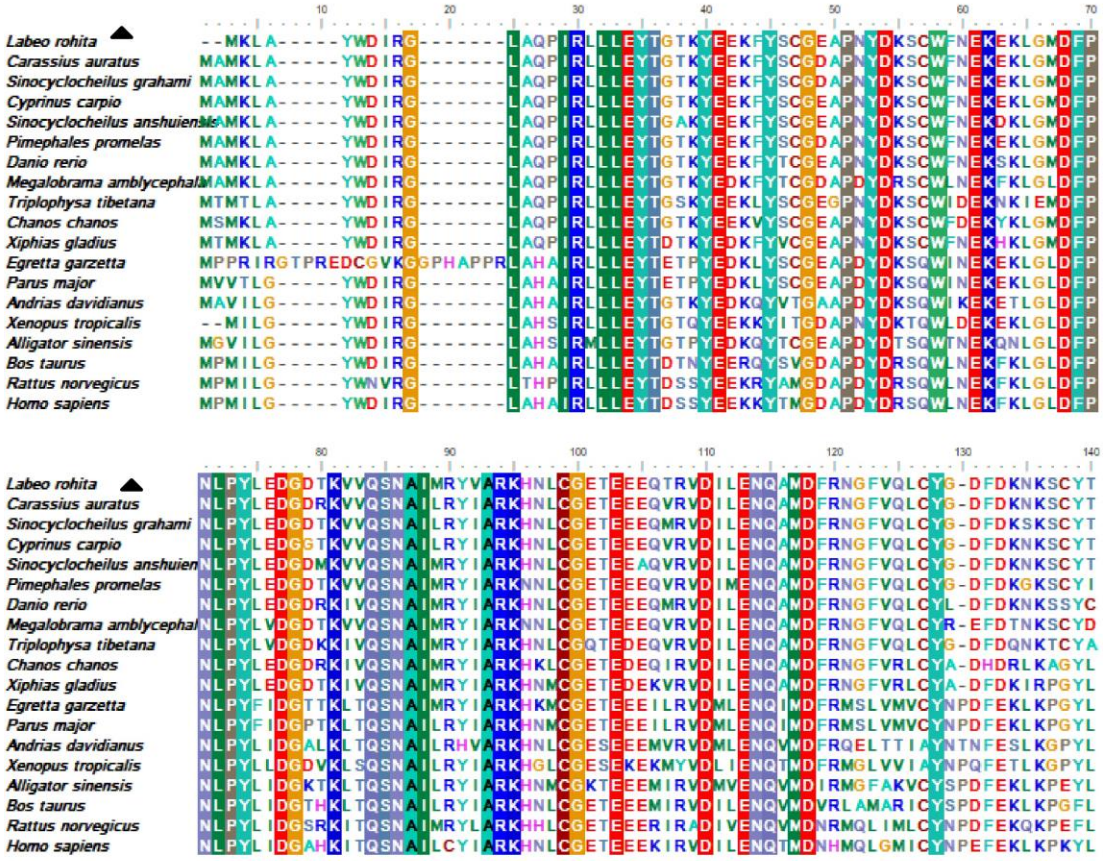

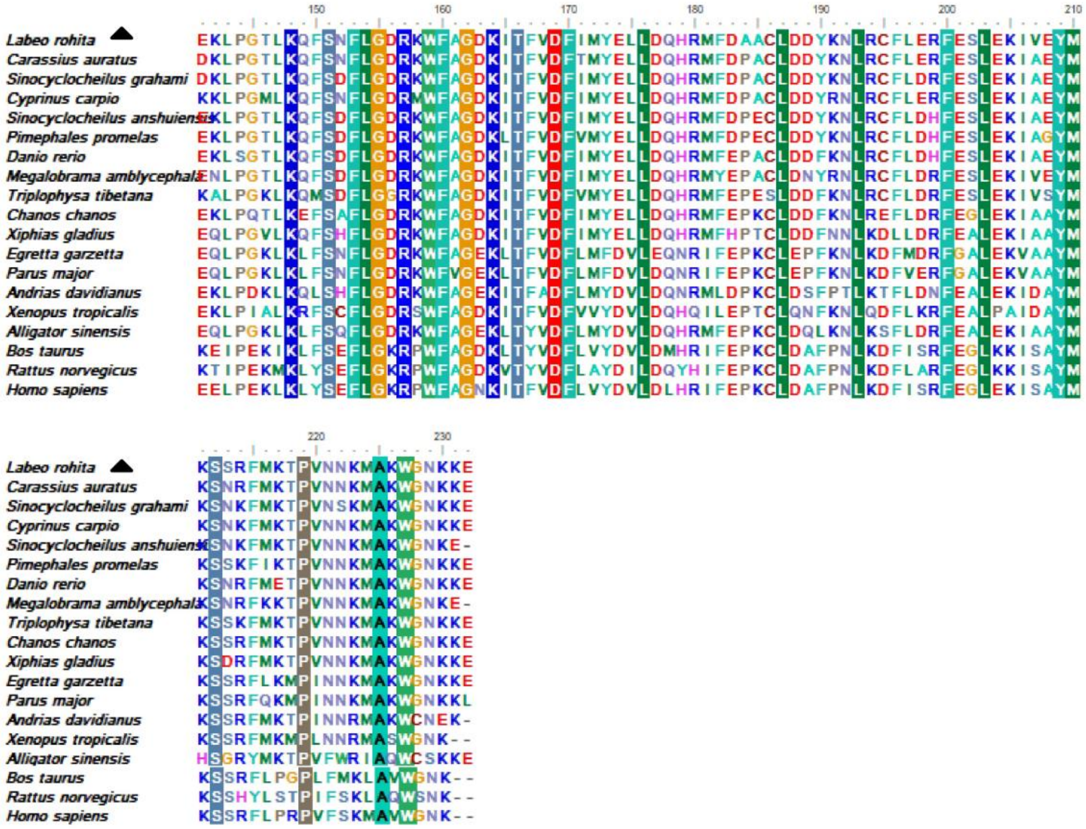

Figure S4. Alignment of *Lr*GST mu's amino acid sequences with those of other species.

a

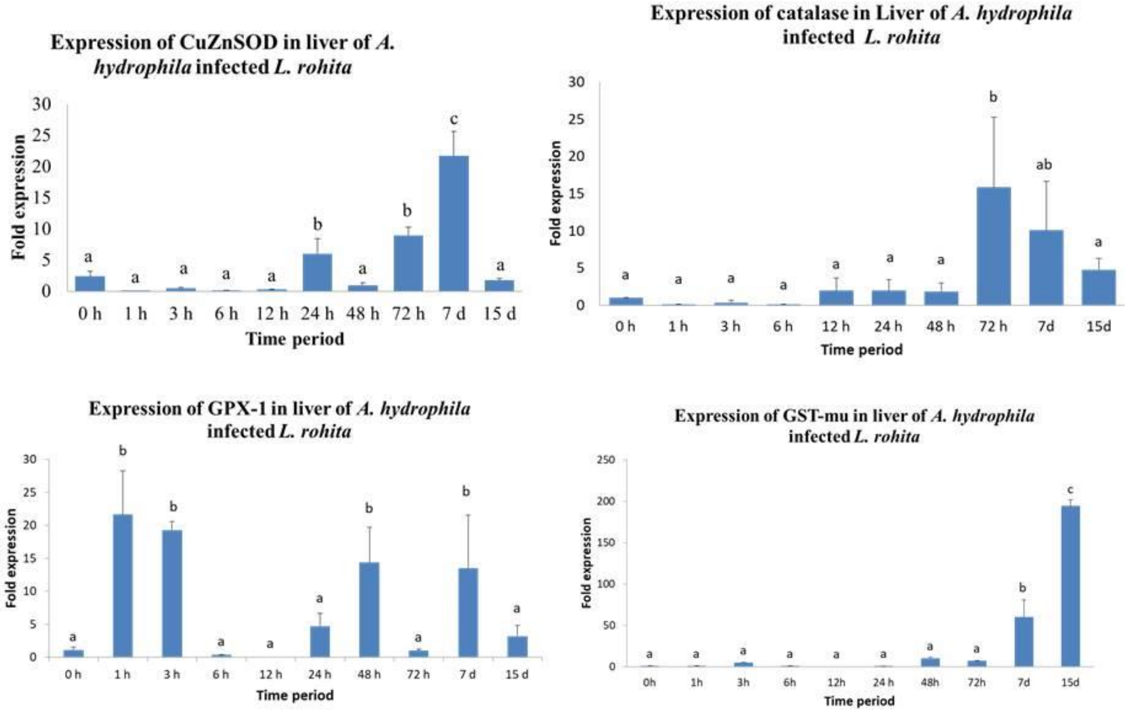

Expression of CuZnSOD in liver of A. hydrophila infected L. rohita
Fold expression
Time period
0 h 1 h 3 h 6 h 12 h 24 h 48 h 72 h 7 d 15 d
Expression of catalase in Liver of A. hydrophila infected L. rohita
Fold expression
Time period
0 h 1 h 3 h 6 h 12 h 24 h 48 h 72 h 7d 15d
Expression of GPX-1 in liver of A. hydrophila infected L. rohita
Fold expression
Time period
0 h 1 h 3 h 6 h 12 h 24 h 48 h 72 h 7 d 15 d
Expression of GST-mu in liver of A. hydrophila infected L. rohita
Fold expression
Time period
0h 1h 3h 6h 12h 24h 48h 72h 7d 15d

b

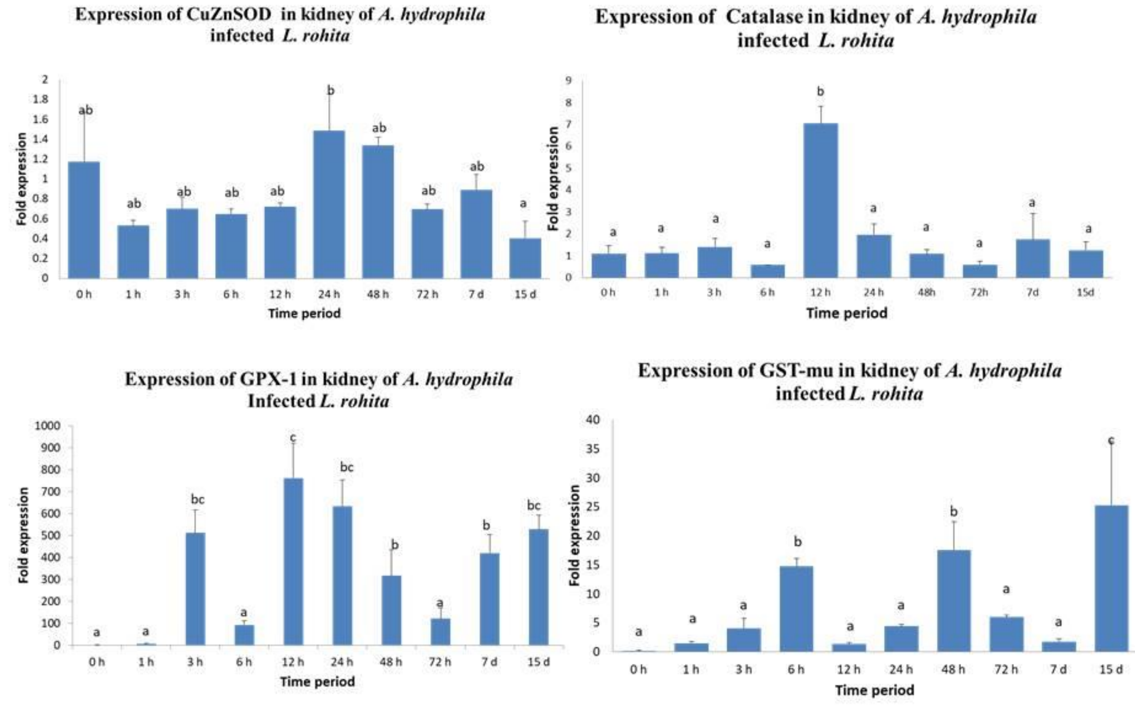

Expression of CuZnSOD in kidney of A. hydrophila infected L. rohita
Fold expression
Time period
0 h 1 h 3 h 6 h 12 h 24 h 48 h 72 h 7 d 15 d
Expression of Catalase in kidney of A. hydrophila infected L. rohita
Fold expression
Time period
0 h 1 h 3 h 6 h 12 h 24 h 48h 72h 7d 15d
Expression of GPX-1 in kidney of A. hydrophila Infected L. rohita
Fold expression
Time period
0 h 1 h 3 h 6 h 12 h 24 h 48 h 72 h 7 d 15 d
Expression of GST-mu in kidney of A. hydrophila infected L. rohita
Fold expression
Time period
0 h 1 h 3 h 6 h 12 h 24 h 48 h 72 h 7 d 15 d

c

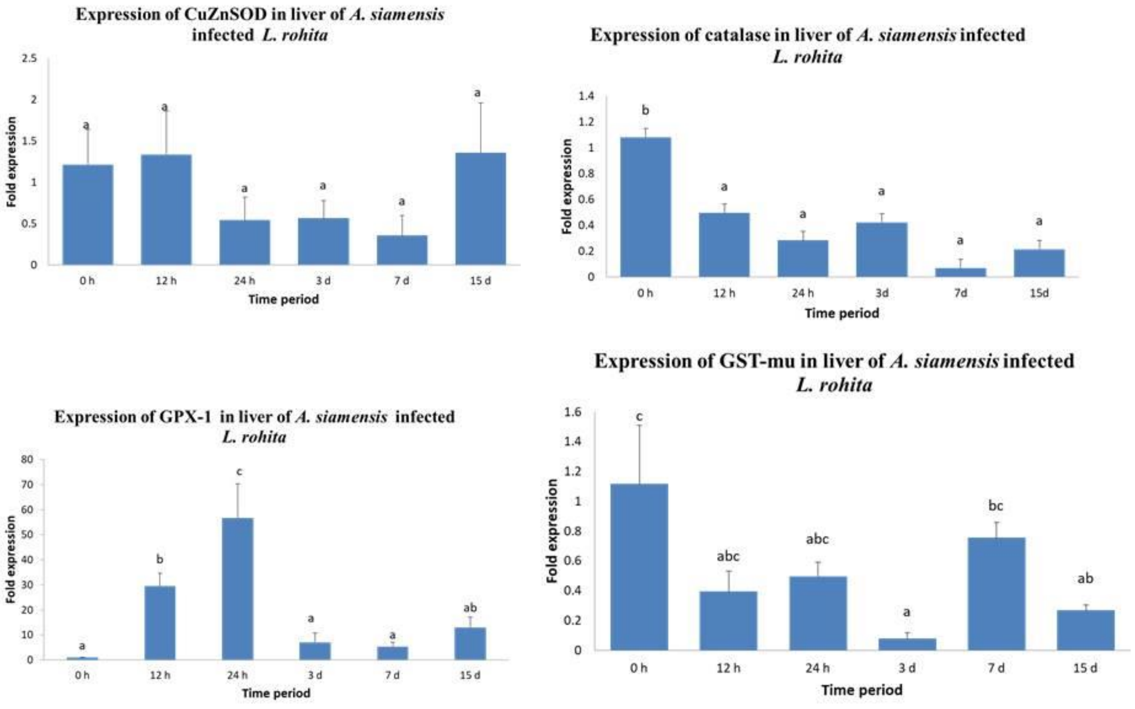
Expression of CuZnSOD in liver of A. siamensis infected L. rohita
Fold expression
Time period
0 h
12 h
24 h
3 d
7 d
15 d
Expression of catalase in liver of A. siamensis infected L. rohita
Fold expression
Time period
Expression of GPX-1 in liver of A. siamensis infected L. rohita
Fold expression
Time period
Expression of GST-mu in liver of A. siamensis infected L. rohita
Fold expression
Time period

d

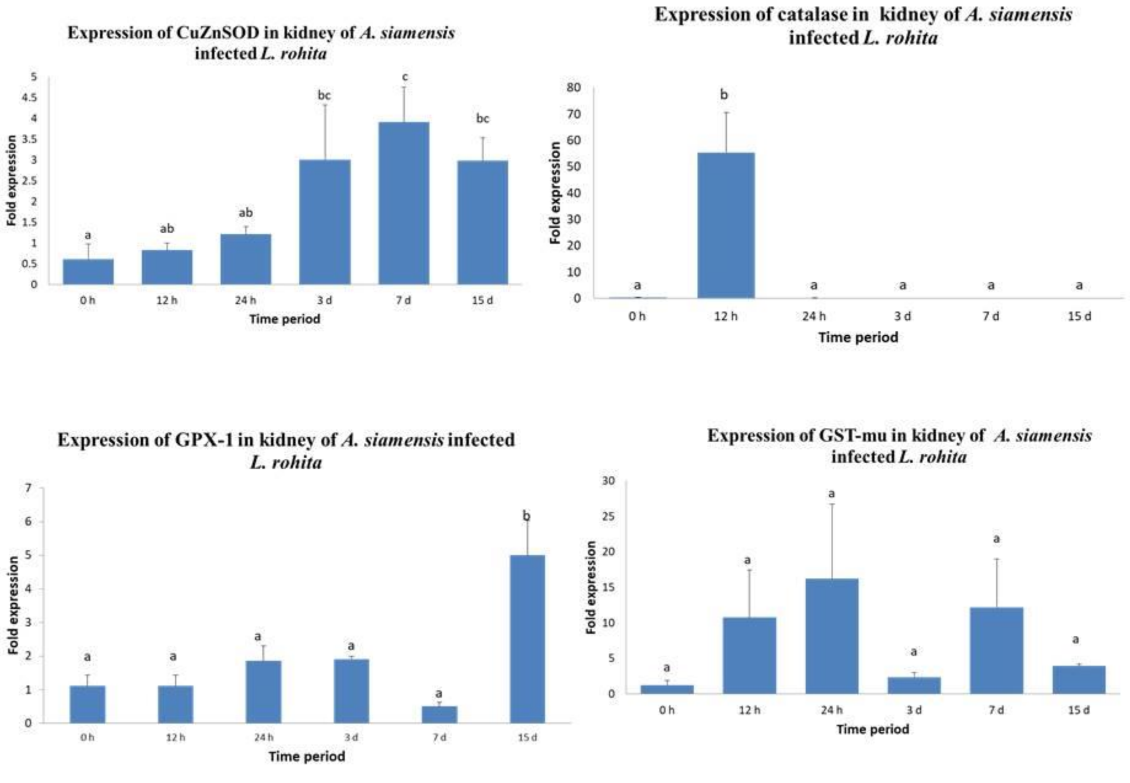
Expression of CuZnSOD in kidney of A. siamensis infected L. rohita
Fold expression
Time period
0 h
12 h
24 h
3 d
7 d
15 d
Expression of catalase in kidney of A. siamensis infected L. rohita
Fold expression
Time period
Expression of GPX-1 in kidney of A. siamensis infected L. rohita
Fold expression
Time period
Expression of GST-mu in kidney of A. siamensis infected L. rohita
Fold expression
Time period

e

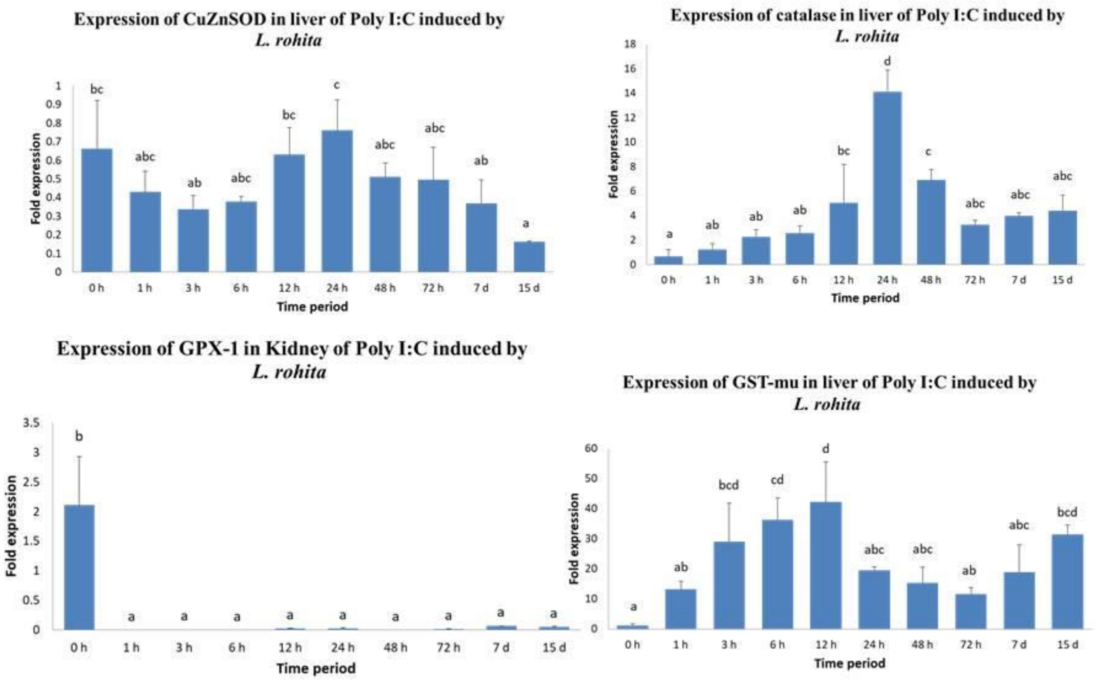
Expression of CuZnSOD in liver of Poly I:C induced by
L. rohita
Fold expression
Time period
0 h
1 h
3 h
6 h
12 h
24 h
48 h
72 h
7 d
15 d
Expression of catalase in liver of Poly I:C induced by
L. rohita
Expression of GPX-1 in Kidney of Poly I:C induced by
L. rohita
Expression of GST-mu in liver of Poly I:C induced by
L. rohita

f

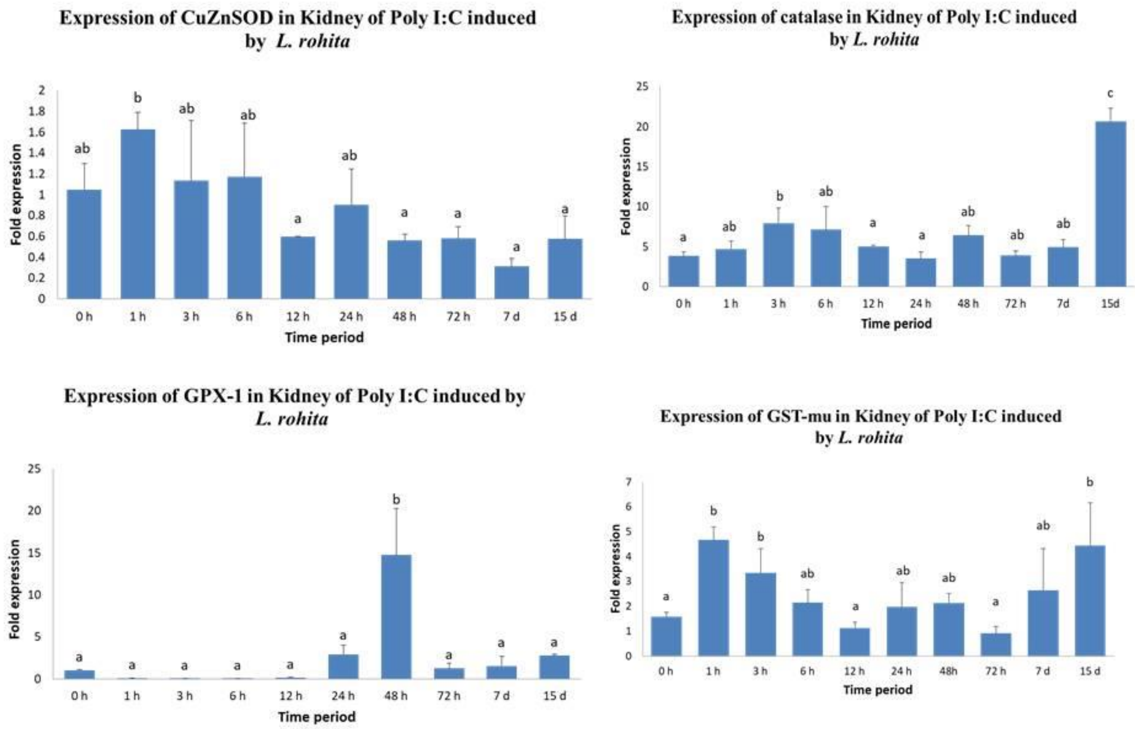
Expression of CuZnSOD in Kidney of Poly I:C induced
by L. rohita
Fold expression
Time period
Expression of catalase in Kidney of Poly I:C induced
by L. rohita
Expression of GPX-1 in Kidney of Poly I:C induced by
L. rohita
Expression of GST-mu in Kidney of Poly I:C induced
by L. rohita

g

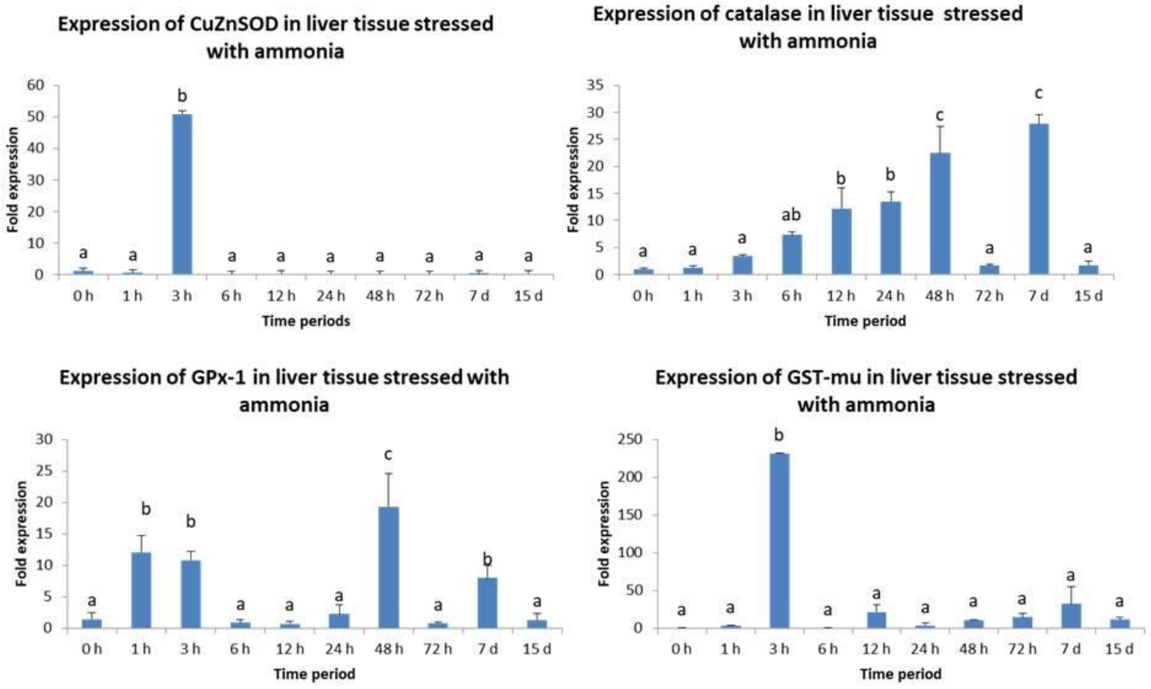
Expression of CuZnSOD in liver tissue stressed with ammonia
Fold expression
Time periods
Expression of catalase in liver tissue stressed with ammonia
Fold expression
Time period
Expression of GPx-1 in liver tissue stressed with ammonia
Fold expression
Time period
Expression of GST-mu in liver tissue stressed with ammonia
Fold expression
Time period
0 h 1 h 3 h 6 h 12 h 24 h 48 h 72 h 7 d 15 d

h

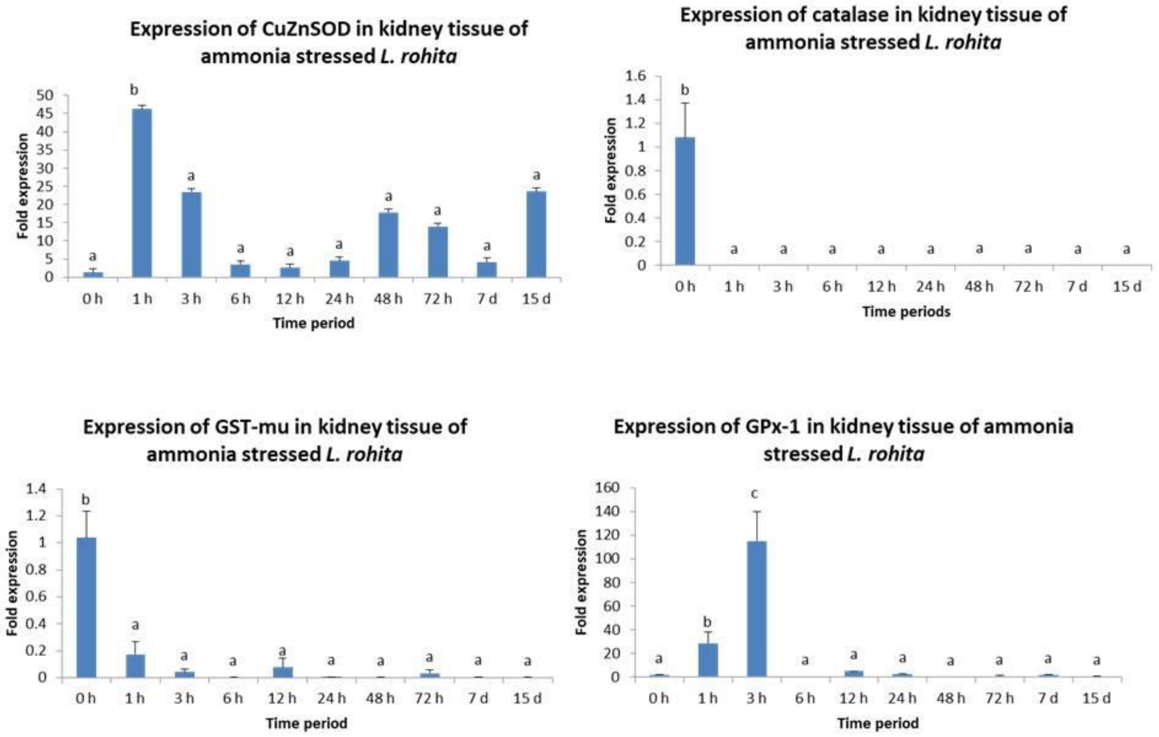
Expression of CuZnSOD in kidney tissue of ammonia stressed *L. rohita*
Fold expression
Time period
Expression of catalase in kidney tissue of ammonia stressed *L. rohita*
Fold expression
Time periods
Expression of GST-mu in kidney tissue of ammonia stressed *L. rohita*
Fold expression
Time period
Expression of GPx-1 in kidney tissue of ammonia stressed *L. rohita*
Fold expression
Time period
0 h 1 h 3 h 6 h 12 h 24 h 48 h 72 h 7 d 15 d

Figure S5(a.b,c,d,e,f,g,h). Expression of GPX-1, GST-mu, CuZnSOD, and CAT genes in *L. rohita* liver and kidney tissues at various time points following *Aeromonas hydrophila* challenge, infection with *Argulus siamensis*, following poly I:C induction and ammonia exposure (h: hours following challenge; d: days following challenge; n = 3). a, b, c, d and e on top of the bar represent significant gene transcript levels (P<0.05) between naive fish and other groups of fish at various time points.

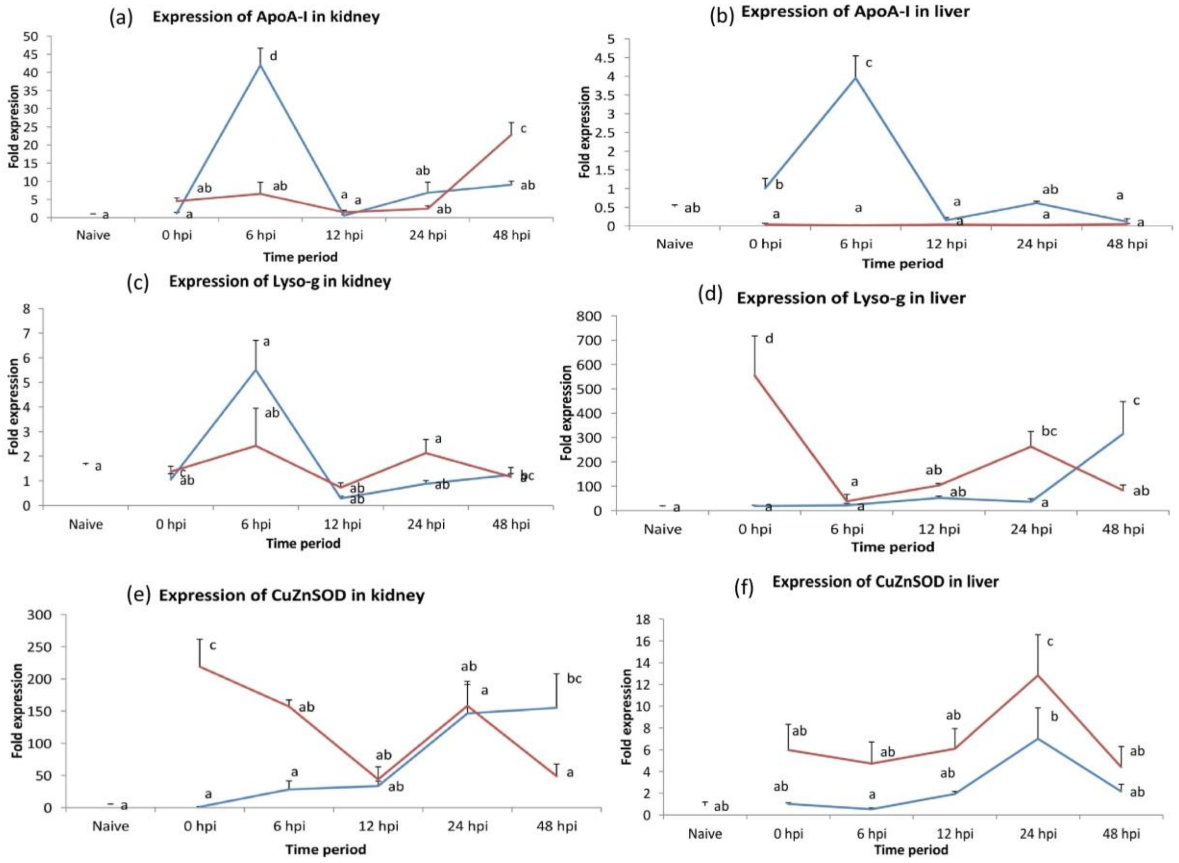

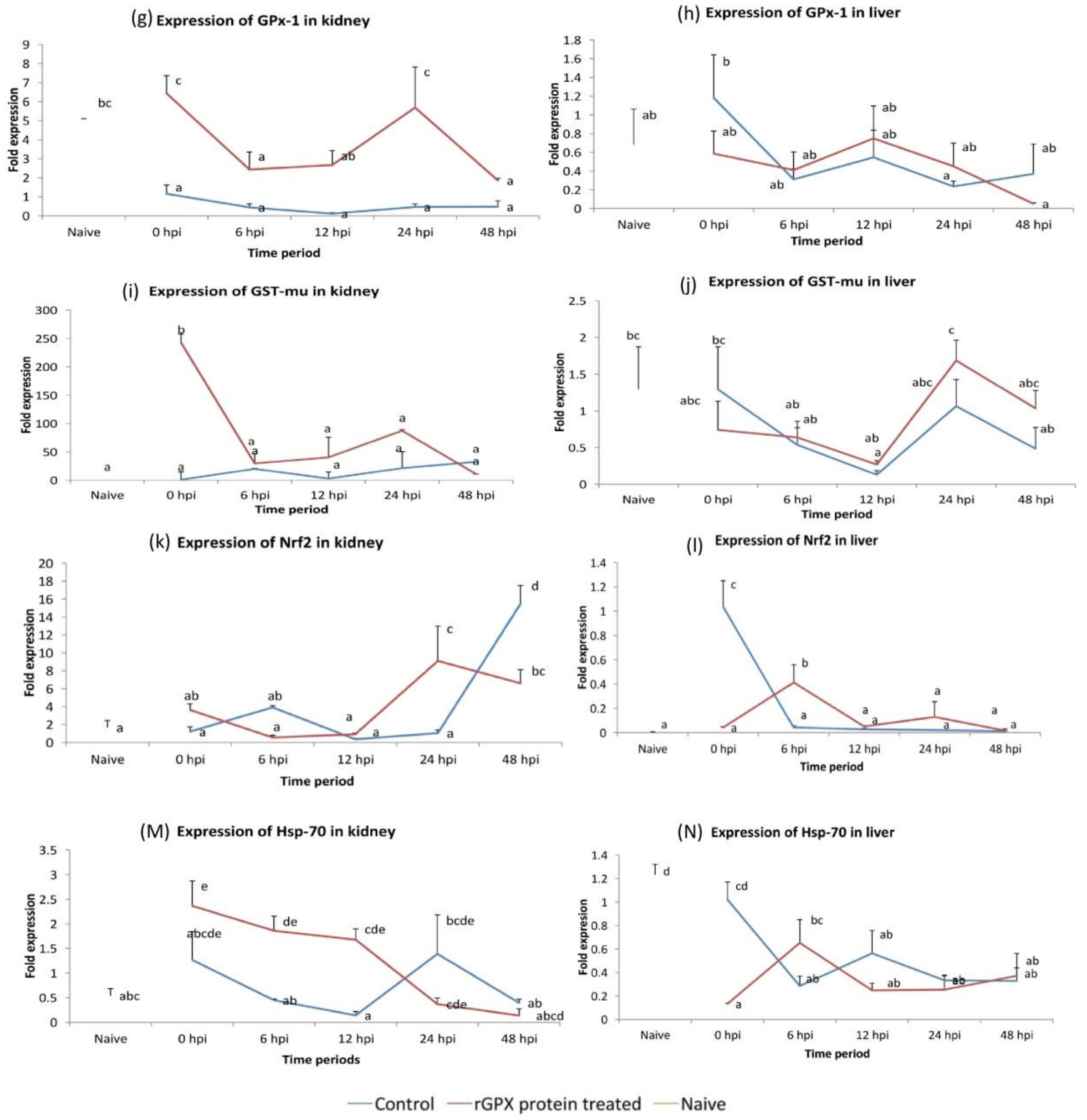

Figure S6. Variation in expression pattern of different immune-related and antioxidant genes, ApoA1 in kidney (a) and liver (b), Lyso-G in kidney (c) and liver (d), CuZnSOD in kidney (e) and liver (f),

GPX-1 in kidney (g) and liver (h), GST-mu in kidney (i) and liver (j), nrf-2 in kidney (k) and liver (l) and hsp-70 in kidney (m) and liver of different groups of *L. rohita* (treated with r*Lr*GPX-1) at different time periods (n = 3). The bars show the mean ± S.E. of three fish. a, b, c, d and e on top of the bar represent significant gene transcript levels (P<0.05) between naive fish and other groups of fish at various time points.

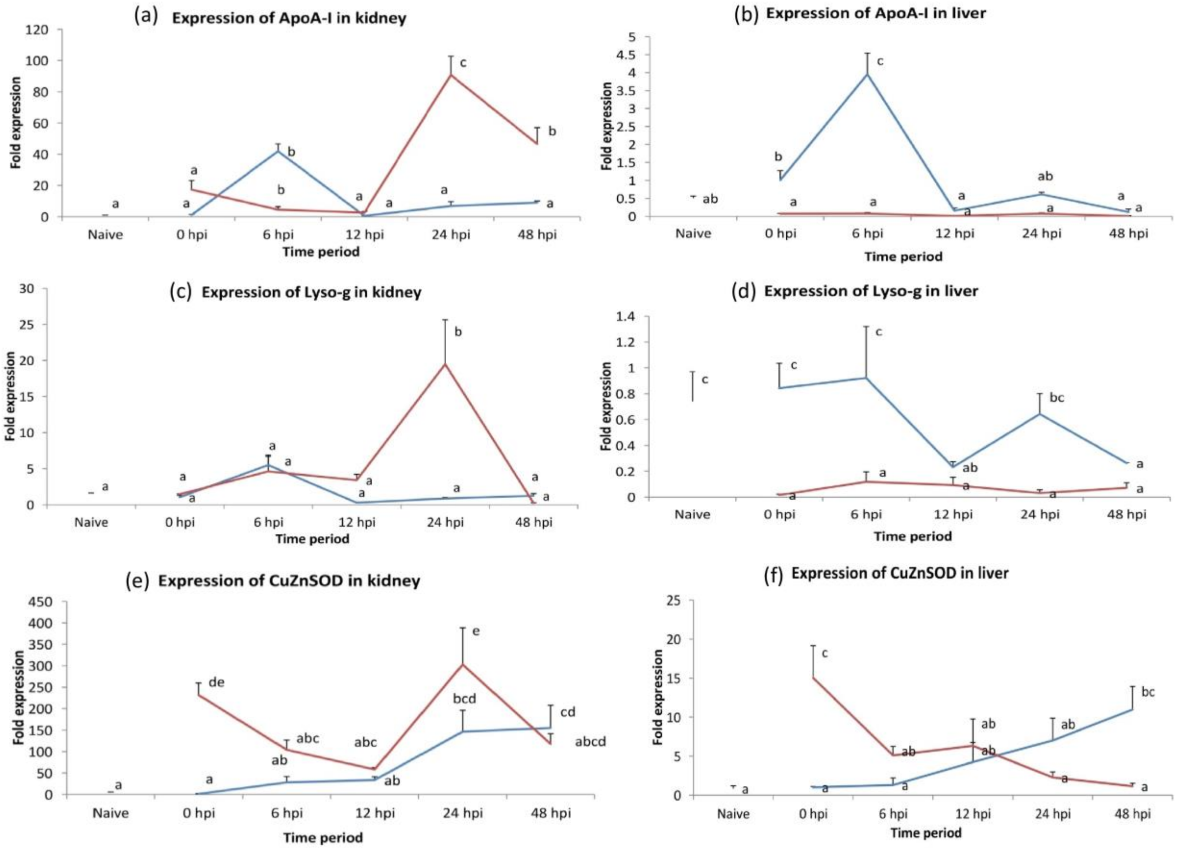

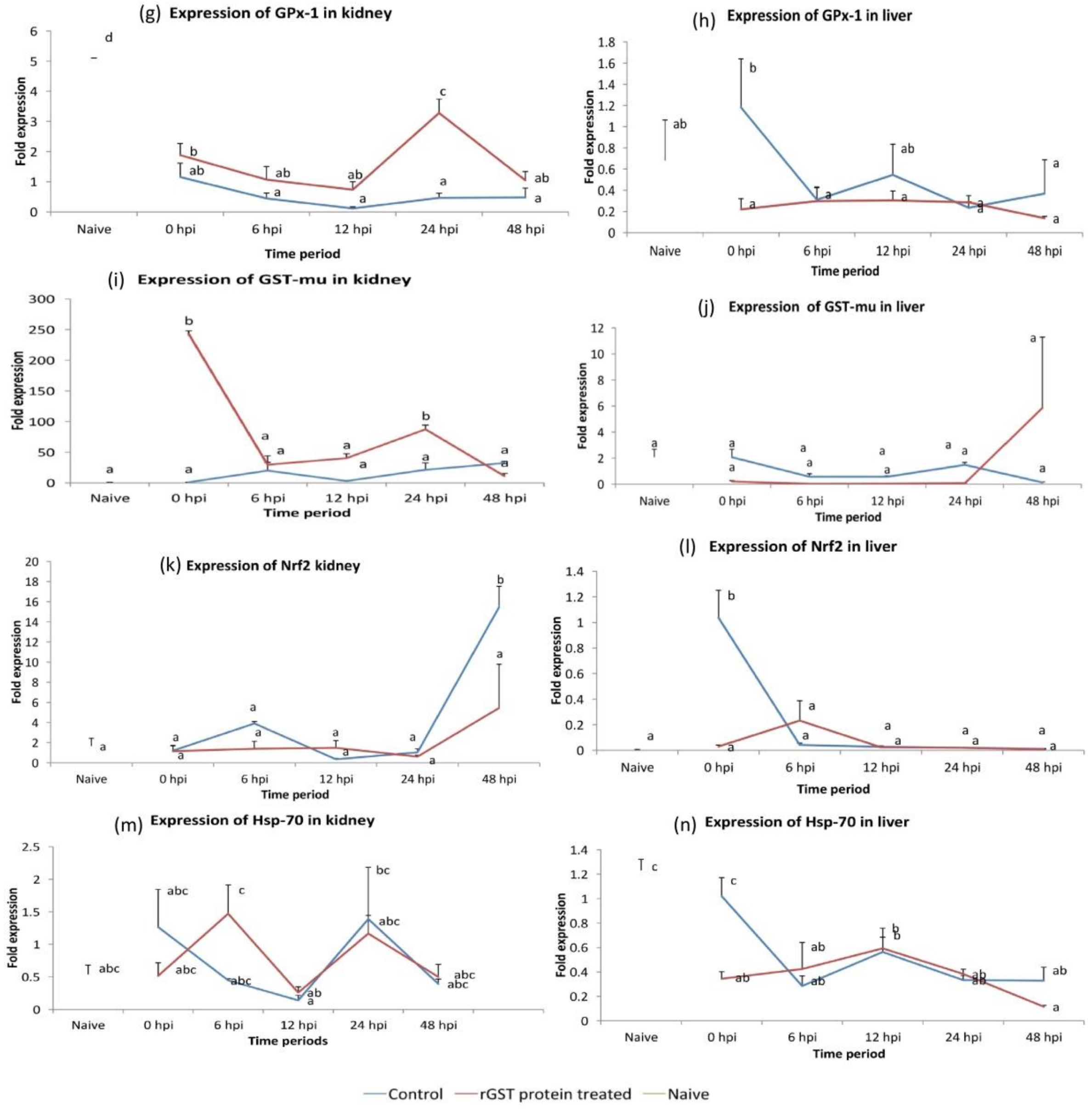

Figure S7. Variation in expression pattern of different immune-related and antioxidant genes, ApoA1 in kidney (a) and liver (b), Lyso-G in kidney (c) and liver (d), CuZnSOD in kidney (e) and liver (f),

GPX-1 in kidney (g) and liver (h), GST-mu in kidney (i) and liver (j), nrf-2 in kidney (k) and liver (l) and hsp-70 in kidney (m) and liver of different groups of *L. rohita* (treated with r*Lr*GST-mu) at different time periods (n = 3). The bars show the mean ± S.E. of three fish. a, b, c, d and e on top of the bar represent significant gene transcript levels (P<0.05) between naive fish and other groups of fish at various time points.

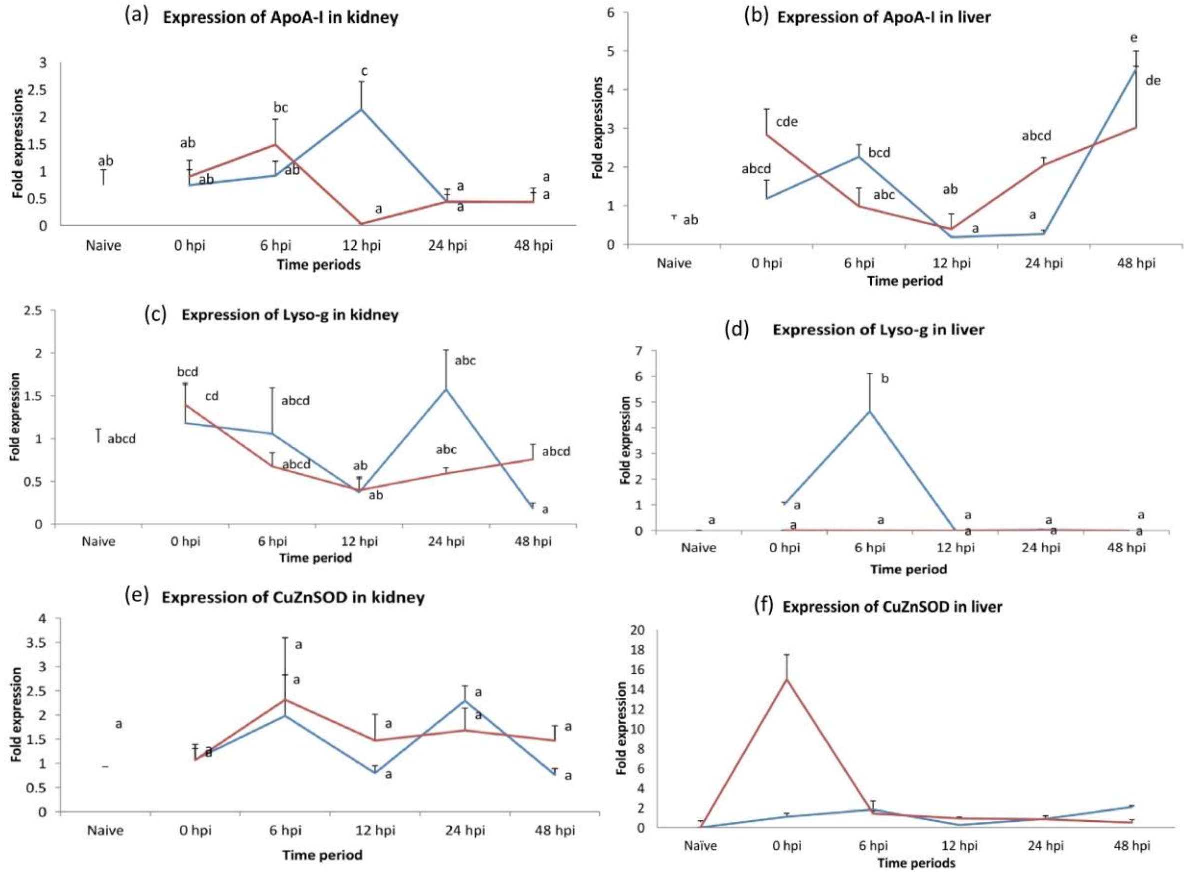

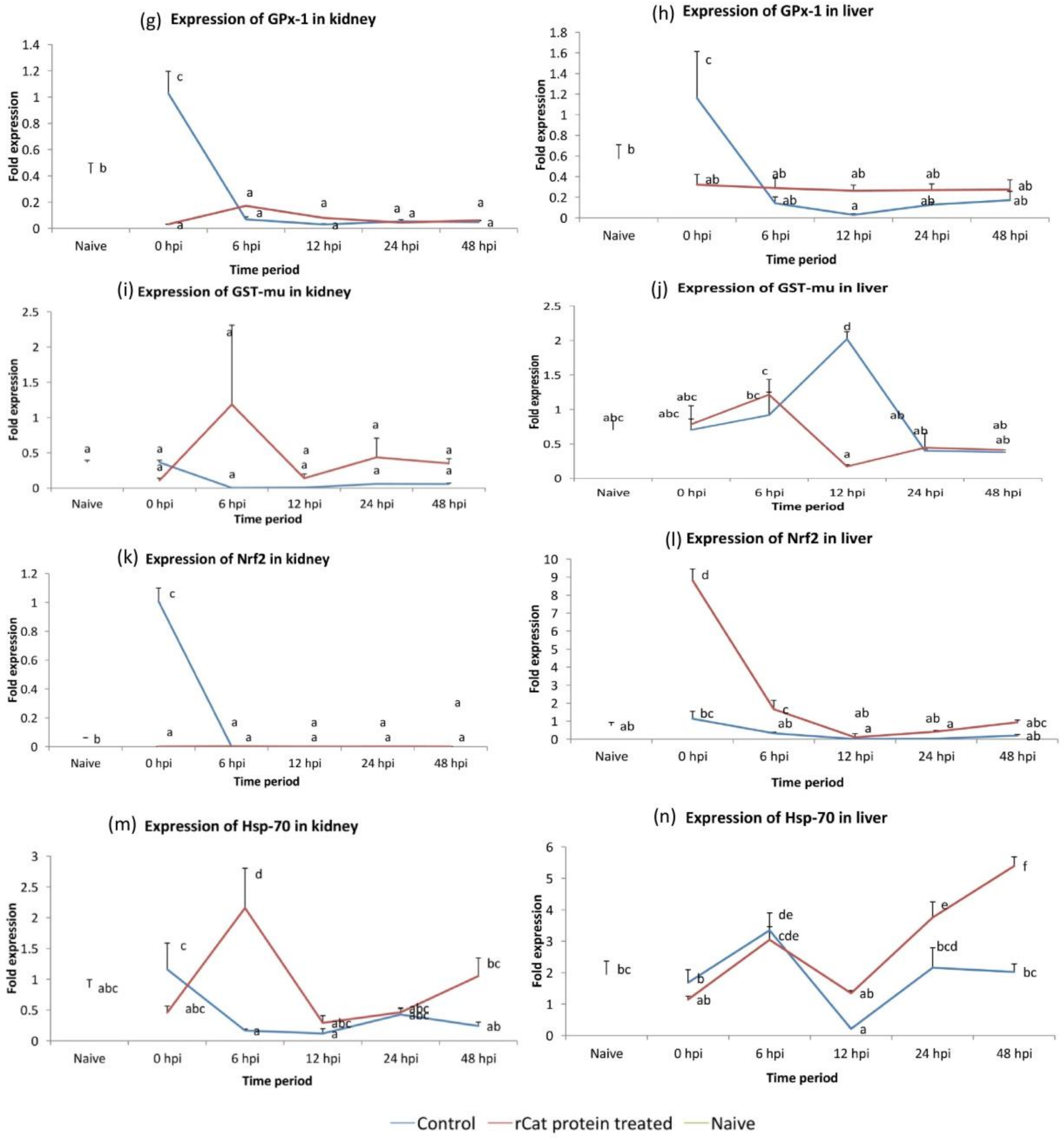

Figure S8. Variation in expression pattern of different immune-related and antioxidant genes, ApoA1 in kidney (a) and liver (b), Lyso-G in kidney (c) and liver (d), CuZnSOD in kidney (e) and liver (f),

GPX-1 in kidney (g) and liver (h), GST-mu in kidney (i) and liver (j), nrf-2 in kidney (k) and liver (l) and hsp-70 in kidney (m) and liver of different groups of *L. rohita* (treated with r*Lr*CAT) at different time periods (n = 3). Bars represent mean ± S.E of three fish. a, b, c, d, e and f on top of the bar represent significant gene transcript levels (P<0.05) between naive fish and other groups of fish at various time points.

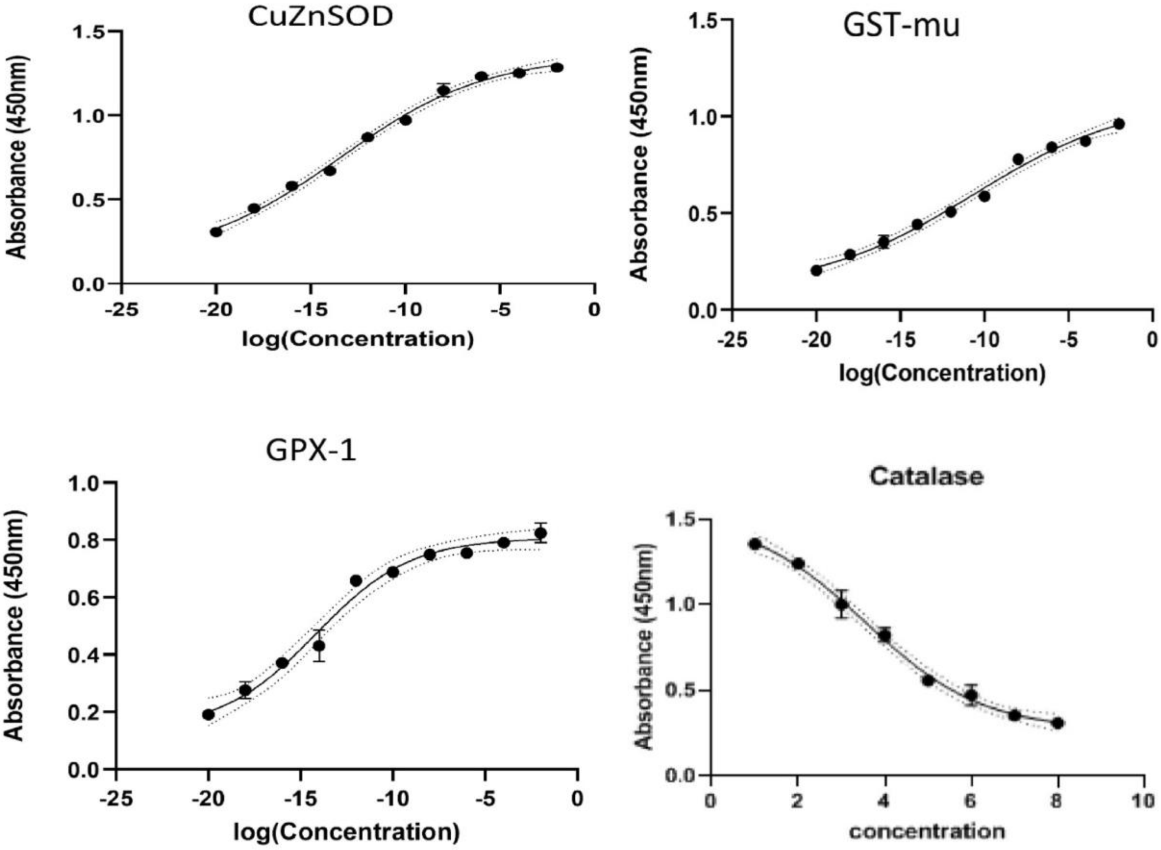

Figure S9. Standard curve used for quantification of normal serum CuZnSOD, GPX-1, GST-mu and CAT level in *L. rohita*.
